# Integrated gut microbiota and metabolomics analysis reveals the antitumor effects of ergosta-4,6,8(14),22-tetraen-3-one purified from the medicinal fungus pholiota adiposa in tumor-bearing mice

**DOI:** 10.3389/fphar.2025.1653035

**Published:** 2025-12-16

**Authors:** Boyuan Liu, Junsen Qin, Shan Zheng, Jiaxin Zhang, Xuemei Hu, Xiaoyan Wang, Yan Liu

**Affiliations:** 1 Academy of Traditional Chinese Medicine, Jilin Agricultural Science and Technology University, Jilin, China; 2 Academy of Medical, Changchun Sci-Tech University, Changchun, China

**Keywords:** ergosta-4, 6, 8(14), 22-tetraen-3-one, metabolomics, gut microbiota, H22 tumor-bearingmice, pharmacological mechanism

## Abstract

**Objective:**

This study aims to investigate the antitumour mechanism of ergosta-4,6,8(14),22-tetraen-3-one (ETO), extracted from Pholiota adiposa, in H22 tumour-bearing mice through integrated metabolomics and gut microbiota systems analysis.

**Methods:**

After establishing the H22 tumor mouse model, we performed serum metabolomics through nontargeted UHPLC-HRMS and conducted gut microbiota profiling.

**Result:**

ETO exhibited significant antitumor effects, with tumor inhibition rates of 59.74% (low dose) and 76.10% (high dose).Untargeted metabolomics revealed that molecules downstream of the tricarboxylic acid cycle, arachidonic acid and linoleic acid were upregulated. Gut microbiota analysis revealed ETO-mediated enrichment of beneficial bacteria (Firmicutes and Lactobacillus) and suppression of the harmful bacterial species Bartonella. Integrative analysis demonstrated a negative correlation between indole-3-acetonitrile and Bartonella.

**Conclusion:**

ETO can inhibit the growth of tumor cells, and its effect on liver and kidney damage is less than that of CTX. Linoleate, and L-tryptophan emerged as potential therapeutic targets, while Firmicutes and Lactobacillus enrichment suggests gut microbiota-mediated antitumor activity, providing novel insights for hepatocellular carcinoma treatment.

## 1 Introduction

In recent years, cancer has emerged as a major global health threat (Liu et al., 2015; Gall et al., 2019; Fauer et al., 2020). Cancer research plays an indispensable role not only in improving individual health but also in strengthening social stability and security (Li et al., 2025). Hepatocellular carcinoma (HCC) is one of the most prevalent and likewise one of the most lethal malignant tumors in the world, accounting for about 4.1% of the annual incidence of malignant tumors (Shen et al., 2015). HCC is a disease caused by triggering an immune response to its own antigens (Xu et al., 2023). According to the US SEER database, the average 5-year survival rate is 19.6%, but for advanced metastatic cases, it can be as low as 2.5% (Chidambaranathan-Reghupaty et al., 2021). HCC grows in small nodules and often remains asymptomatic for several years. The survival rate of untreated patients mainly depends on the degree of underlying liver function impairment and the size of the tumor at the initial diagnosis (Ryder and British Society of Gastroenterology, 2004). Commonly used cancer treatments include drugs, surgery, radiotherapy, however, these treatments often have limited efficacy and many adverse effects. Cyclophosphamide (CTX) remains one of the most successful and widely utilized agents for investigating the antitumor effects of bioactive compounds (Emadi et al., 2009). Despite its antitumor activity, CTX causes varying degrees of organ damage in human body. Furthermore, chemical anticancer drugs are costly, and their severe adverse effects on the body cause significant patient distress (Renata et al., 2022). Therefore, there is an urgent need to explore and evaluate novel therapeutic agents with antitumor effects and minimal hepatorenal toxicity. Natural plant compounds have long been an important source of drug treatment, supporting the field of drug discovery and development (Miao et al., 2025). Currently, substantial evidence indicates that steroids derived from edible and medicinal mushrooms demonstrate good promising anticancer effects (Konstantinos et al., 2020). A previous study indicated that ergosta-4, 6, 8(14), 22-tetraen-3-one (ETO) effectively inhibits HepG-2 cell proliferation *in vitro* and exhibits anti-H22 tumor activity *in vivo* (Wang et al., 2020). Although ETO has shown promising results in tumor therapy, its underlying mechanism of action remains unclear. Therefore, this study aimed to elucidate the mechanism of the antitumor effects of ETO.

Ergosterol is the primary chemical precursor of steroids. It occurs extensively in edible and medicinal fungi and serves as a precursor complex for vitamin D synthesis. This significance of ergosterol is due to its biological profile as well as the challenges associated with its precise synthesis because of its complex stereochemistry (Reguera et al., 2019; Lin et al., 2017). According to previous literature, ergosterol from edible and medicinal fungi exhibits anti-inflammatory properties and reduces cytokine release (Daniel et al., 2020).

Pholiota adiposa is native to Northeast Asia and is an edible fungus of extremely high value. Its fruiting bodies have strong anti-tumor activity. It is an excellent source of polysaccharides, proteins, sterols and vitamins (Jatuwong et al., 2020). With significant medicinal value, the fruit body of *P. adiposa* is a source of numerous functional compounds, such as polysaccharides, peptides, polyunsaturated fatty acids, ribonucleic acids, lectins, triterpenoids, adenine, and steroids. P. adiposa consumption offers potential benefits, including effective regulation of the human body and heat dissipation (Zou et al., 2019). Therefore, it is critical to elucidate the therapeutic effects of ergosterol derived from P. adiposa in tumor treatment. This study was designed and implemented to address this objective.

In recent years, research on metabolomics and gut microbiota has emerged as a rapidly developing discipline following genomics, transcriptomics, and proteomics, and has been widely used in many fields, such as animal and plant metabolism (Trivedi et al., 2017; Yan et al., 2020), microbial metabolism (Martin et al., 2012), disease diagnosis (Prejac et al., 2020), and drug development (Zhu et al., 2021). Metabolomics, the targeted and untargeted analysis of small molecule metabolites (<1,500 Da) from endogenous and exogenous sources, is being increasingly utilized to identify metabolites associated with disease treatment or diagnosis of diseases, and it may provide a better pathophysiological understanding of diseases (Aihua et al., 2012). By conducting metabolomics studies, the antitumor mechanism of ETO is investigated by examining its regulation of physiological and pathological metabolism, with a focus on metabolites and metabolic pathways.

The impact of the gut microbiota on health and disease is increasingly evident, with studies suggesting that the microbiota functions as a biological ecosystem that interacts strongly with the host, while the host genome influences the gut microbiome (Herbert et al., 2018; Serino et al., 2009). Therefore, the present study conducted fecal 16S ribosomal RNA sequencing to comprehensively understand the entire genome of the intestinal microflora, including the relationship between intestinal and pathogenic bacterial strains.

In the present study, we further investigated the antitumor activities of ETO, derived from P. adiposa, and its mechanisms in H22 hormonal mice. Subsequently, we examined ETO-mediated regulation of gut microbiota and metabolic processes and conducted an integrated gut microflora and metabolomics analysis to establish the correlation between them. We sought to elucidate ETO’s tumor-suppressing effects and underlying mechanisms to support its further development and application.

## 2 Materials and methods

### 2.1 Materials and reagents

The fruits of P. adiposa were purchased from Songyuan City, Changchun, China, which was identified by Professor Tolgor Bau of Jilin Agricultural University as Pholiota adiposa. All chemical reagents used in this study were analytical grade which were purchased from Merck (Darmstadt, Germany). Deionized water was purified by Milli-Q water purification system (Millipore, Billerica, MA, United States). Nanjing Jiancheng Bioengineering Research Institute (China) supplied the Hematoxylin and Eosin (H&E) staining kits used in this study. TUNEL assay was performed *in situ* by using the apoptosis detection kit (Roche, Branchburg, NJ, United States) and the DAB detection kit. Interleukin-2 (IL-2), Interleukin-6 (IL-6), Tumor Necrosis Factor-α (TNF-α), Interferon-gamma (IFN-γ), VEGF, Alanine aminotransferase (ALT), Aspartate aminotransferase (AST), Blood urea nitrogen(BUN)and Cyclization Recombination Enzyme (CRE) ELISA kits were purchased from American R&D Co., Ltd. (Minneapolis, MN, United States). Cyclophosphamide (CTX) for injection was purchased from Jiangsu Shengdi Pharmaceutical Co., Ltd. (Nanjing, China). *In situ* cell death detection kit (Roche Applied Science, Switzerland). All Rabbit polyclonal antibodies and mouse monoclonal antibody for Western blot were purchased from Cell Signaling Technology (Danvers, MA, United States).

Enhanced Chemiluminescence Detection System (Amphamasia Biotech); Light microscope, DMi 8 inverted fluorescence microscope, RM2015 embedding machine (Leica, Germany); MiniSpin centrifuge (Eppendorf AG, Germany); YD- 1508R Rotary Slicer (Jinhua Yidi Medical Equipment Co., Ltd.); SZC- 101 Intelligent Automatic Soxhlet Extractor (Shanghai Fiber Inspection Instrument Co., Ltd.).

### 2.2 Preparation of ETO

**FIGURE 1:**
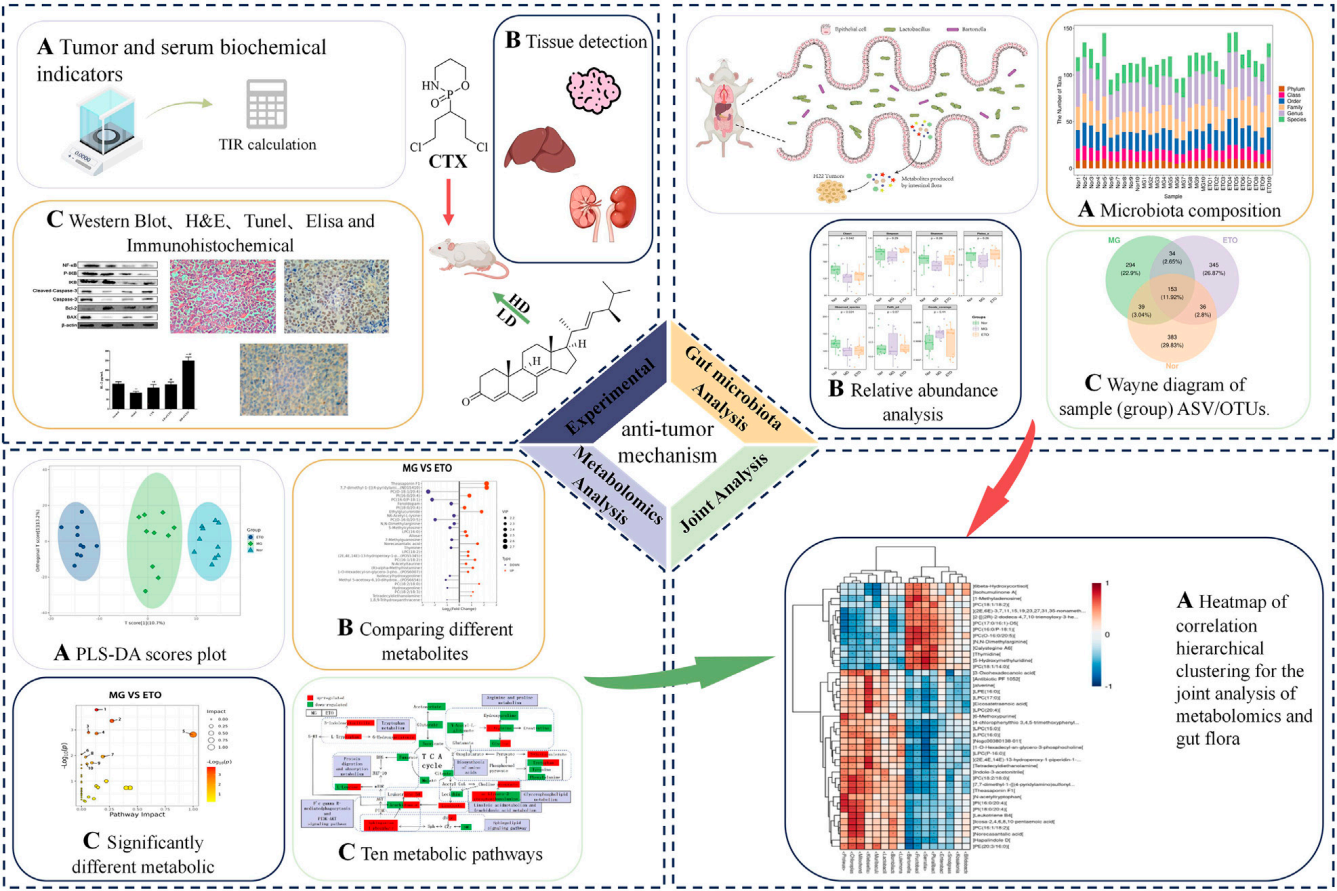
The mechanism diagram of the anti-tumor effect of ETO.

The 1.5 kg fruit body of P. adiposa was dried, crushed, and extracted with 95% ethanol under reflux at 60 °C to obtain the ethanol extract, which was further processed with petroleum ether (60 °C–90 °C) to yield the petroleum ether fraction. This fraction was subjected to silica gel column chromatography to obtain the petroleum: chloroform mixture at ratios of 75:1, 50:1, 25:1, and 10:1. The compound ETO was isolated from the 50:1 petroleum ether: chloroform fraction (Figure 1A). The extraction process was performed by referring to a previous method. Structural analysis of ETO was conducted by 1H NMR (600 MHz) and 13C NMR (125 MHz) spectroscopy at the Changchun Institute of Applied Chemistry, Chinese Academy of Sciences (Figures 1B,C). Subsequent experiments used chromatography grade 98% ETO purchased from Baoji Chenguang Biotechnology Co. in Shaanxi Province, China.

### 2.3 Determination of *in vivo* antitumor activity

Male ICR mice (specific pathogen-free grade, 6–8 weeks old, weight: 20 ± 2 g) were obtained from Liaoning Changsheng Biotechnology Co., Ltd. (Liaoning, China) (Certificate No.: SCXK (Liao) 2023-0001). The animals received standard laboratory diet and water *ad libitum* and were maintained at 25 °C ± 2 °C with a 12-h light/dark cycle (lights on: 8:00 a.m. to 8:00 p.m.). All mice underwent environmental adaptation for 1 week. The experimental procedures strictly adhered to the Regulations of Experimental Animal Administration issued by the Ethical Committee for Laboratory Animals at Jilin Agricultural University (Permit No. ECLA-JLAU-23056).

**FIGURE 2:**
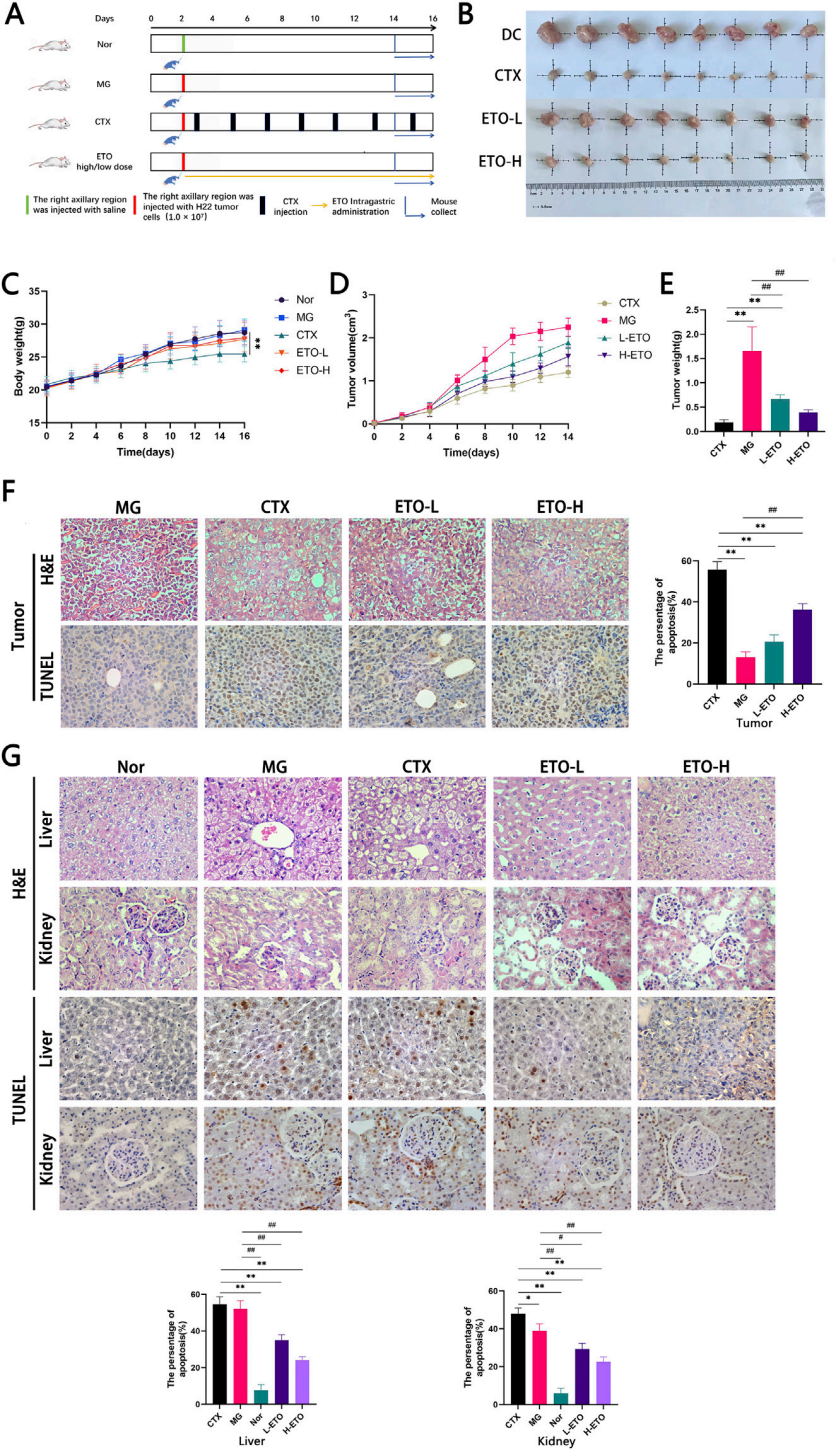
ETO inhibited the growth of the H22 tumor. **(A)** Timeline of animal experiments. **(B)** Tumor images of different treated groups at the termination of animal experiment in the tumor mouse model of H22 cells. **(C)** Changes in bodyweight during drug intervention. Data were expressed as means ± SD, n = 10. **p < 0.01 Compared with Nor group. **(D,E)** Changes in bodyweight, tumor volume, and tumor weight during drug intervention. **(F)** H&E stained sections of the tumor and TUNEL assay results. **(G)** H&E stained sections and TUNEL assay of the liver and kidney results. L-ETO: ETO at 0.05 mmol/kg. H-ETO: ETO at 0.1 mm g/kg. CTX: CTX at 25 mg/kg.

The H22-hepatoma cell line (mouse origin) was obtained from Jilin Provincial Cancer Hospital (Jilin, China). Following established protocols, we prepared H22 tumor-bearing mice by maintaining the tumor cells in ascitic form through serial passages in the peritoneal cavities of female Balb/c mice. To avoid a series of immune rejections that may be caused by allogeneic transplantation, ascites tumor cells from female mice were inoculated into male mice. The specific operation is as follows: After preparing the H22 liver cancer cell suspension (1.0 × 10^7^ cells/mL), we established the tumor model by performing a 0.1 mL subcutaneous injection in the right axillary region of all experimental mice, except for the normal group (Nor). After 24 h, 10 mice in each group were randomly divided into five groups. The experimental design is shown in Figure 2A. We classified the experimental animals as follows: untreated tumor-bearing mice (model group), CTX-treated tumor-bearing mice (positive treatment group, CTX), and ETO-treated tumor-bearing mice (ETO treatment group). The ETO group was further divided into high-dose (39.262 mg/kg; ETO-H) and low-dose (19.631 mg/kg; ETO-L) groups(MW = 392.62). The Nor group was administered saline solution intragastrically in equivalent volumes, whereas the positive control group received 25 mg/kg CTX intraperitoneally. Treatments were performed once daily for 14 consecutive days. Afterward, all mice were euthanized, and tumor tissues were excised and weighed.

### 2.4 Calculation of mouse body weight, tumor volume, and tumor suppression rate and estimation of biochemical parameters from mice serum

The tumor inhibition rate (TIR) was calculated using the formula: 100% × (average tumor weight of the control group - average tumor weight of the treatment group)/average tumor weight of the control group. The derivative exhibiting the highest TIR was selected for subsequent experiments. Following 14 days of treatment, the mice were euthanized, and the tumor blocks were extracted, weighed, and photographed. The tumor inhibitory index (TIR) was calculated using the formula: 100% × (average tumor weight of the model group - average tumor weight of the treatment group)/average tumor weight of the model group. Fresh blood samples were acquired from the orbital venous plexus of the mice and heparinized. The blood samples were centrifuged at 1,500 rpm for 20 min at 4 °C, and the sera were collected and stored at 4 °C for biochemical assay. The levels of interleukin-2 (IL-2), IL-6, tumor necrosis factor-α (TNF-α), interferon-γ (IFN-γ), vascular endothelial growth factor (VEGF), alanine aminotransferase (ALT), aspartate aminotransferase (AST), blood urea nitrogen (BUN), and creatinine (CRE) in each group were measured using ELISA kits according to the specified protocols (Deng et al., 2017).

### 2.5 Tissue assays

Hematoxylin and eosin (H&E) staining of H22 solid tumor cells was performed using the respective kit in accordance with the manufacturer’s instructions. The results were examined using an optical microscope. H&E-stained tumor tissue sections were imaged at 400× for morphological analysis (Fischer et al., 2008). TUNEL assay was conducted to detect apoptotic cells in tumor tissues (Han et al., 2019). Cell morphology was observed using a Zeiss AXIO Scope A1 microscope. Subsequently, 50 μL TUNEL solution was added, and the cells were incubated at 37 °C for 60 min. Peroxidase activity in each tissue section was confirmed by applying diaminobenzidine. The tumor tissues were fixed with 10% formaldehyde solution for 24 h, subjected to routine decolorization, paraffin-embedded, and cut into 4-µm-thick slices. Immunohistochemical staining was performed using a murine/rabbit IgG immunohistochemistry kit (Danvers, MA, United States).

### 2.6 Western blotting assay

After freezing the tissue samples, protein hydrolase, phosphatase inhibitor, lysis solution, and EDTA were added for lysis. The samples were homogenized and incubated for 30 min. Subsequently, the mixture was centrifuged at 12,000 g for 30 min at 4 °C. The supernatant was collected, quantified, and boiled at 100 °C for 10 min. The resulting solution was then analyzed by SDS-PAGE, and the separated proteins were transferred to PVDF membranes. The membranes were then blocked with 5% skimmed milk for 2 h at room temperature and then incubated overnight with the following antibodies: anti-BAX (1:1000), anti-Bcl-2 (1:1000), anti-caspase-3 (1:1000), anti-cleaved-caspase-3C (C-caspase-3) (1:1000), anti-IKB (1:1000), anti-P-IKB (1:1000), anti-NF-κB (1:1000) and anti-β-actin (1:2000). After rinsing with TBST, the membranes were incubated with secondary antibodies for 1 h, developed using a developing solution (Clinx, Shanghai, China), and characterized using ImageJ software (National Institutes of Health, Maryland, United States). β-Actin was used as the internal reference gene.

### 2.7 Metabolomics analysis

We added 100 μL of 80% methanolic aqueous solution to 100 μL of serum sample. Following vigorous shaking and vortexing, samples underwent 5-min ice-bath cooling prior to centrifugation (15,000 × g, 15 min, 4 °C). An aliquot of supernatant was collected, adjusted to 53% methanol with MS-grade water, and centrifuged (15,000 g, 4 °C, 15 min). The supernatant was collected and subjected to ultra-high performance liquid chromatography (UHPLC)-MS/MS analysis. Raw data files generated by UHPLC-MS/MS were processed using Compound Discoverer 3.1 (CD3.1, Thermo Fisher, United States) for peak alignment, peak selection, and quantification of each metabolite. MS analysis was performed using a Thermo Vanquish UHPLC system equipped with a Thermo Hypersil Gold column (100 × 2.1 mm, 1.9 μm) coupled to a Thermo QE series mass spectrometer (both from Thermo Fisher Scientific). The offline data files were processed using CD-Search software (CD3.1). Equal volumes of samples from each experimental sample were mixed and used as the QC sample. The elution program was as follows: 0–2 min: 98% A, 30% B; 2–5 min: 70% A, 30% B; 5–8 min: 20% A, 80% B; 8–8.1 min: 10% A, 90% B; and 8.1–10 min: 90% A, 10% B. The MS parameters were as follows: scan range: m/z 100–1500, ESI source spray voltage: 3.5 kV, sheath gas flow rate: 35 psi, gas flow rate: 35 psi, auxiliary gas flow rate: 10 L/min, capillary temperature: 320 °C, and S-lens radiofrequency (RF) level: 60. Kyoto Encyclopedia of Genes and Genomes (KEGG) enrichment analysis was conducted on significantly different metabolites. Parameters such as retention time and mass-to-charge ratio were screened, and peak alignment was performed with a retention time deviation of 0.2 min and mass deviation of 5 ppm to further improve the accuracy of identification. The peaks were then extracted according to the following set criteria: mass factor: 5 ppm, signal intensity deviation: 30%, signal-to-noise ratio: 3, minimum signal intensity: 100,000, and added ions; this was followed by peak area quantification and target ion integration. Molecular formulae were predicted and compared with the McCloud database. A blank sample was used to remove background ions, and a quality control sample was used to normalize the quantitative results for data identification and quantitative results. Peaks were matched against the McCloud (https://www.mzcloud.org/), Mzvault, and MassList databases to obtain accurate qualitative and relative quantitative results. Statistical analyses were performed using statistical software R (version 3.4.3), PYTHON (version 2.7.6), and CentOS (version 6.6). The regional normalization method was applied for non-normally distributed data.

### 2.8 Gut microbiota analysis

Total DNA was extracted using the Fast DNA Spin Kit for Soil in strict accordance with the manufacturer’s instructions. PCR for the extracted DNA was performed using the KAPA HiFi Hotstart ReadyMix PCR Kit on an ABI GeneAmp 9700 PCR instrument (ABI, Foster City, CA, United States) with the following primers for the V3-V4 hypervariable regions of the 16S ribosomal RNA (rRNA) gene: 338F (5’-ACTCCTACGGGAGGCAGCAG-3’) and 806R (5’-GGACTACHVGGGTWTCTAAT-3’). The amplicons were purified and quantified before sequencing on the HiSeq 2500 PE250 Amplifier sequencing platform (Illumina, San Diego, CA, USA) by a commercial company (Bioprofile Technology Co., Ltd., Shanghai, China). The original sequences that passed the initial quality screening were sorted into libraries and samples according to index and barcode information, and the barcode sequences were then removed. Sequence denoising clustering was then performed using the QIIME 2 DADA2 plugin. The alpha-diversity level of each sample was assessed based on the distribution of amplicon sequence variant (ASV) in different samples, and the sparse curve reflected the appropriateness of the sequencing depth. At the ASV level, the distance matrix of each sample was calculated, and inter-group differences in beta-diversity and significance were measured by a variety of unsupervised sorting and clustering methods combined with the corresponding statistical tests. To elucidate the taxonomic composition of species, several unsupervised and supervised methods of ranking, clustering, and modeling, combined with appropriate statistical tests, were utilized to further measure differences in species abundance composition between different samples (groups) and to find appropriate marker species. Based on the compositional distribution of species across samples, association networks were constructed, topological indices were calculated, and key species were identified. Next, based on the 16S rRNA gene sequencing results, the metabolic function of the sample’s flora was predicted, the differential pathways were identified, and the species composition of specific pathways was obtained. Finally, based on the above results, graphs were plotted and analyzed with statistical tests.

### 2.9 Statistical analysis

Data were analyzed and plotted using GraphPad Prism 6.0.1 software (GraphPad Software, San Diego, United States) and IBM SPSS Statistics R26.0.0.0 software (IBM, New York, NY, United States). Data are expressed as mean ± standard deviation (SD). Statistical differences between groups were determined using one-way analysis of variance (ANOVA). P-value <0.05 and P-value <0.01 were considered statistically significant. For qualitative and quantitative analysis of metabolites, data preprocessing was conducted using CD 3.1 software. Following sample pretreatment, molecular and fragment ion peaks were analyzed for formula prediction by cross-referencing with the mzCloud, mzVault, and MassList databases. Background subtraction was performed using blank samples, followed by data normalization. Following data identification and quantification, we performed comprehensive metabolomic analyses including quality control assessment, unsupervised dimensionality reduction, OPLS-DA modeling, univariate analysis, volcano plot visualization, and biomarker screening using the MetaboAnalystR package in R program. Metabolites with a P-value of <0.05 or <0.01 were considered differential metabolites. The 16S rRNA sequencing data were statistically analyzed using QIIME2 software (version 2019.10). Differences in microbial community structure between samples were assessed based on the characteristic sequence level alpha-diversity (α-diversity) index and beta-diversity (β-diversity) index and then visualized through principal coordinate analysis and nonmetric multidimensional scaling graphs. Linear discriminant analysis effect size and LDA score were used to identify bacterial subgroups and samples with varying abundances. Taxa with an LDA score of ≥2 were considered significantly different. Based on the relative abundance of major microbial species in the samples, a symbiosis analysis was conducted using Spearman’s correlation coefficient to understand the association between species. PICRUST 1.1.4 software was employed to predict the function of the microbiota and to analyze inter-group functional differences.

## 3 Results

### 3.1 Structural determination

According to literature (Ryder and British Society of Gastroenterology, 2004), the compound is Ergosta-4, 6, 8(14), 22-tetraen-3-one (Supplementary Figures S1A–C).

### 3.2 Experimental study on the inhibition of H22 cell growth by ETO

According to the established experimental process, this study systematically evaluated the regulatory effect of ETO on the proliferation of liver cancer cells *in vivo* by constructing an animal model of H22 solid tumor (Figure 2A). The mice were humanely euthanized on day 16, and their tumor tissues and organs were isolated, weighed, and photographed (Figure 2B). The tumor inhibition rates in the CTX, ETO-H, and ETO-L groups were 88.68% 76.10% and 59.74%, respectively, and the differences were significant when compared with the model group (P < 0.01). A noteworthy finding is that mouse weight gain was significantly inhibited in the CTX group as compared to that in the Nor group (Figure 2C). Furthermore, significant tumor cell growth inhibition was observed in the ETO-H and CTX groups (p < 0.01) (Figures 2D,E). H&E-stained sections and TUNEL assay of tumor cells following ETO treatment revealed distinct morphological changes (Figure 2F). By H & E staining, the tumor cells in the blank group showed oval or round-like morphology, densely packed, and well grown, with intact and clear tumor tissue structure. In contrast, the treatment groups exhibited scattered tumor cells with notable nuclear consolidation and fragmented necrotic tumor tissues. Compared with the ETO-L group, the effect of ETO-H group on inducing tumor cell necrosis showed a dose-dependent difference, but its effect intensity did not reach the level of CTX positive control group. Apoptosis was detected by TUNEL method to quantitatively evaluate the anti-tumor efficacy of ETO. The experiment used brown-yellow particle deposition as a positive criterion. Quantitative analysis showed that the apoptosis rate of CTX positive control group was the highest (59%), which was significantly higher than that of ETO-H treatment group (37%). Histopathological evaluation showed that based on the results of liver and kidney H & E staining and TUNEL detection, the degree of cell damage in the ETO treatment group was significantly lower than that in the CTX positive control group (Figure 2F). These findings suggest that ETO can effectively inhibit H22 tumor growth while maintaining low toxicity.

**FIGURE 3:**
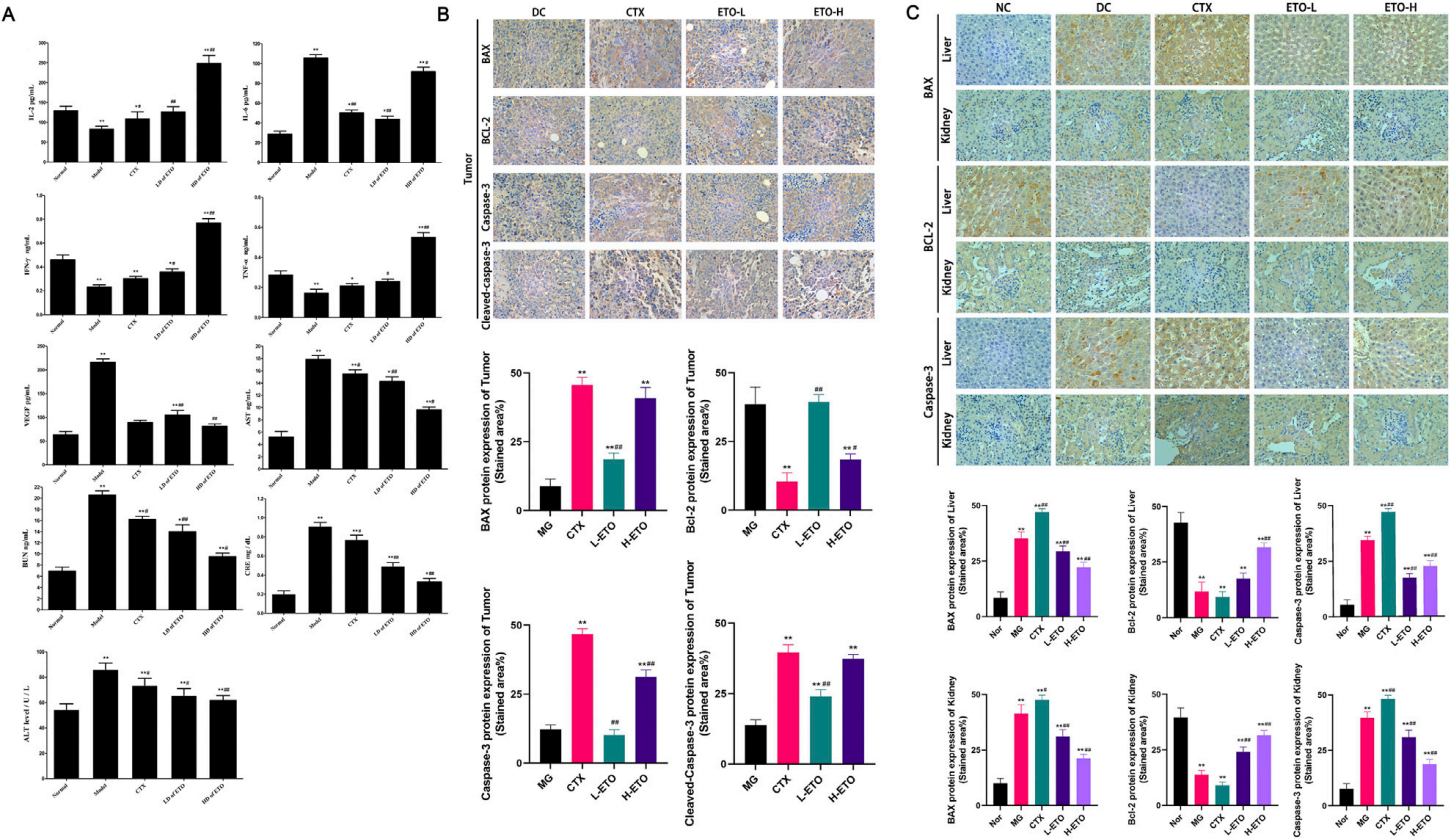
**(A)** Elisa assay for the expression of IL-2, IL-6, TNF-α, IFN-γ, VEGF, ALT, AST, BUN, and CRE in the serum of each group of mice. Data were expressed as means ± SD, n = 3. *p < 0.05 and **p < 0.01 compared with the Nor group. #p < 0.05, ##p < 0.01 compared with the MG group. &p < 0.05 compared with the CAP group. **(B)** Immunohistochemical findings in tumors. **(C)** Immunohistochemical findings in liver and kidney.

According to the results of ELISA, serum IL-2, IFN-γ, and TNF-α levels were significantly elevated in the ETO-H and ETO-L groups as compared to that in the model group. Serum AST, ALT, CRE, and BUN levels were significantly lower in the ETO-H and ETO-L groups than in the CTX group (*p < 0.05 and **p < 0.01, #p < 0.05 and ##p < 0.01) (Figure 3A). To elucidate the antitumor mechanism of ETO, immunohistochemical assay was performed to determine the expression of apoptosis-related proteins, including the antiapoptotic factor Bcl-2 and the proapoptotic factors Bax, caspase-3, and C-caspase-3 (Figure 3B). The results showed that the CTX group exhibited the highest expression levels of BAX, caspase-3, and C-caspase-3 and the lowest expression level of Bcl-2. The ETO-H group showed the second highest expression levels of BAX, caspase-3, and C-caspase-3, and its Bcl-2 expression level was higher than that in the CTX group. To better assess the impact of ETO-induced damage on the liver and kidney, we analyzed the expression levels of Bcl-2, BAX, and caspase-3 in liver and kidney tissues (Figure 3C). The findings indicated lower BAX and caspase-3 expression levels in the liver and kidney tissues of the ETO-H group as compared to that in the CTX group, while the Bcl-2 expression level was higher in the ETO-H group than in the CTX group.

**FIGURE 4:**
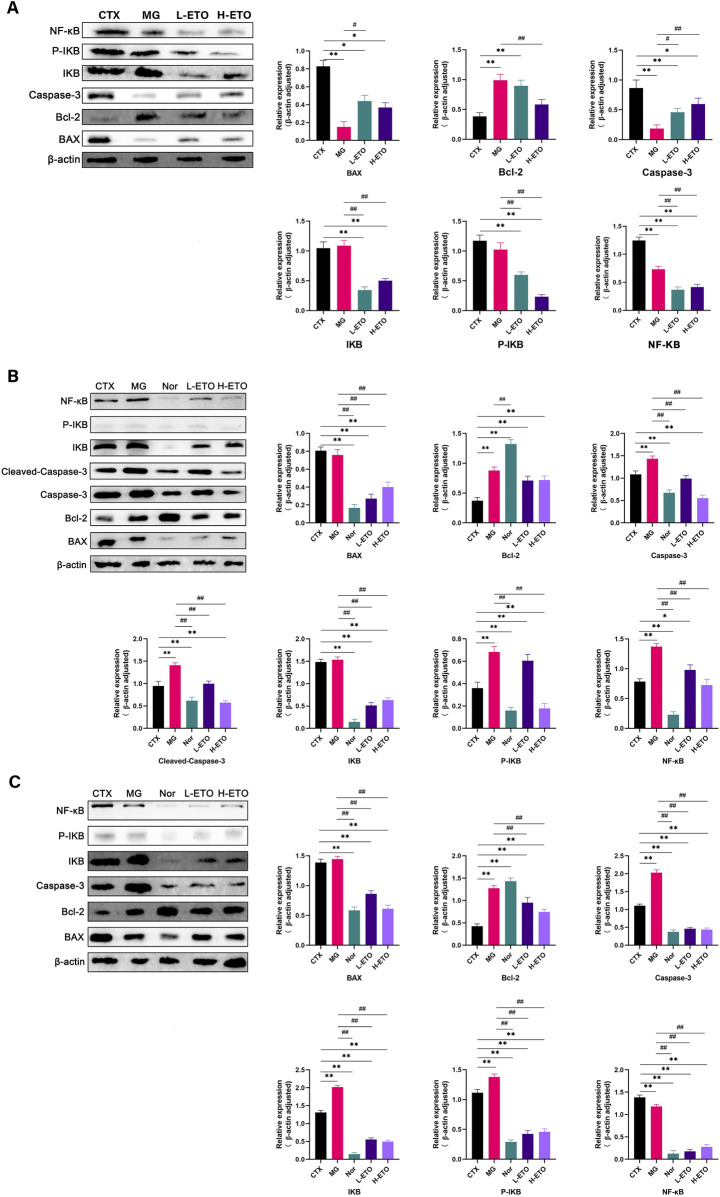
The relative expression of BAX, Bcl-2, Caspase-3, C-Caspase-3, IKB, P-IKB, and NF-κB was analyzed by Western blot. **(A)** Western blot results of the tumor. **(B)** Western blot results of the liver. **(C)** Western blot results of the kidney. Data were expressed as means ± SD, n = 3. *p < 0.05 and **p < 0.01 compared with the CTX group. #p < 0.05, ##p < 0.01 compared with the MG group.

Western blotting assay revealed elevated expression levels of BAX, caspase-3, C-caspase-3, and NF-κB in the ETO-H group as compared to those in the model group; in contrast, the expression levels of Bcl-2, IKB, and P-IKB were decreased in the ETO-H group as compared to those in the model group. This finding indicated that ETO exerted an antitumor effect at a high dose (Figure 4A). The results of Western blotting assay for liver proteins showed higher expression levels of caspase-3 and C-caspase-3 in the ETO-H group than in the CTX group; however, the expression level of BAX in the ETO-H group was significantly lower than that of the CTX group. Additionally, the expression level of Bcl-2 was significantly lower in the ETO-H group than in the CTX group (Figure 4B). The results of immunoblotting assay for kidney proteins showed that the expression levels of caspase-3 and C-caspase-3 were lower in the ETO-H group than in the CTX group; however, the expression levels of BAX and Bcl-2 were higher in the ETO-H group than in the CTX group (Figure 4C).

### 3.3 Stability experiments of serum untargeted metabolomics methods

The histomorphological features were evaluated by H & E staining; TUNEL assay; expression levels of IL-2, IL-6, IFN-γ, VEGF, TNF-α, CRE, AST, ALT, and BUN; and immunohistochemical and Western blotting assays of tumor and liver/kidney tissues, the ETO-H group exhibited second highest antitumor efficacy following the CTX group, with reduced liver/kidney damage. Therefore, the ETO-H group was selected for subsequent intestinal microbiota and metabolomics analyses.

**FIGURE 5:**
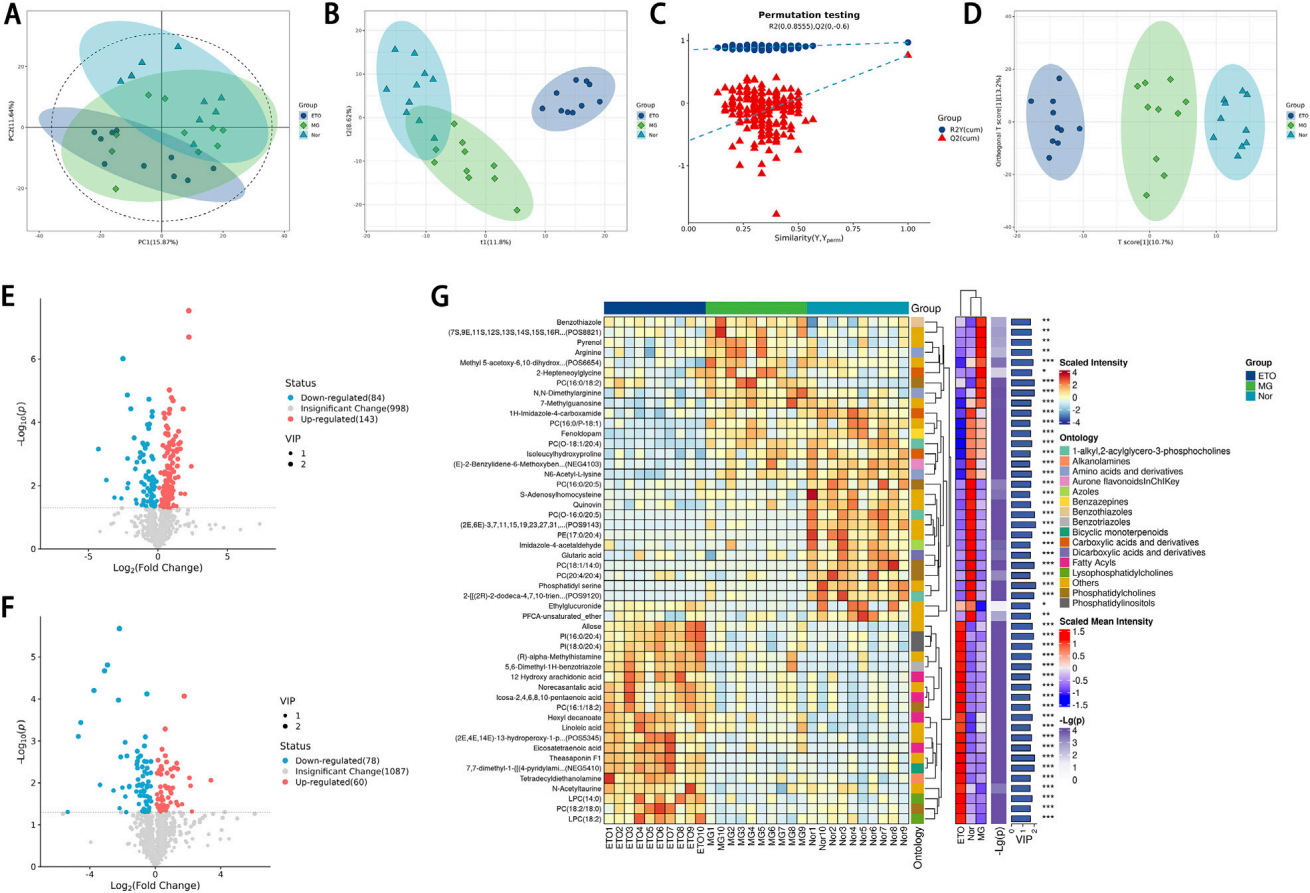
Principal component and partial least-squares discriminant analysis of different groups samples. **(A,B)** PCA scores plot of serum from all mice. **(C)** Corresponding validation plot from ETO, MG and Nor. QC: Quality control. **(D)** PLS-DA scores plot. **(E,F)** Volcano plot indicating upregulated and downregulated metabolites. **(G)** Heat map showing hierarchical clustering of ETO, MG, and Nor mice (top 50 differential metabolites between MG and ETO). Each fruiting bodies sample is visualized in a single column and each metabolite is represented by a single row. Blue colors indicate lower metabolite concentration, while red colors show enhanced metabolite levels. (For interpretation of the references to color in this figure legend.)

Principal component analysis showed that the samples in the ETO group were largely separated from those in the Nor group. The clusters of metabolites in the ETO group showed greater proximity to those in the model group (Figure 5A). The PLS-DA score plot demonstrated separation among the three groups, indicating significant differences in metabolites (Figure 5B). In the PLS-DA permutation test plot, the model parameters of the permutation analysis showed an upward trend, with R2 and Q2 approaching 1 and Q2 exceeding 0.5, thereby confirming the stability and predictive accuracy of the model (Figure 5C). The OPLS-DA score plot exhibited distinct clustering of phenotypes without overlap across the three groups, indicating that the samples were well represented and comparable (Figure 5D).

### 3.4 Screening for differential metabolites

The volcanogram visualization based on univariate statistical analysis data showed the distribution characteristics of differential metabolites between different experimental groups. Combined with the significancant thresholds of variable importance in projection (VIP) value >1 and p-value <0.05 for OPLS-DA variables in the projection, 227 differential metabolites were screened between the model and ETO groups (Figure 5E), and 138 differential metabolites were screened between the Nor and model groups (Figure 5F).

In this study, a hierarchical clustering model was constructed by using the system biology strategy to verify the reliability of the target metabolites and to clarify the correlation between samples and the heterogeneity of metabolic profiles. This approach facilitated accurate screening of marker metabolites and investigation of alterations in associated metabolic processes. Figure 5G shows the top 50 differential metabolites with VIP values. The clustering results on the samples can test the expression stability of the screened target metabolites in the group samples. Additionally, metabolites clustered in the same cluster have similar expression patterns and may be involved in closely related reaction steps during metabolism. Hierarchical clustering of differential metabolite levels in the comparison group. As shown in the figure, the red color scale corresponds to a high expression level, and the blue area is significantly negatively correlated with the low expression level. The metabolite ontology classification information is also presented in Figure 5G.

### 3.5 Functional analysis of differential metabolites and metabolic pathways

**FIGURE 6:**
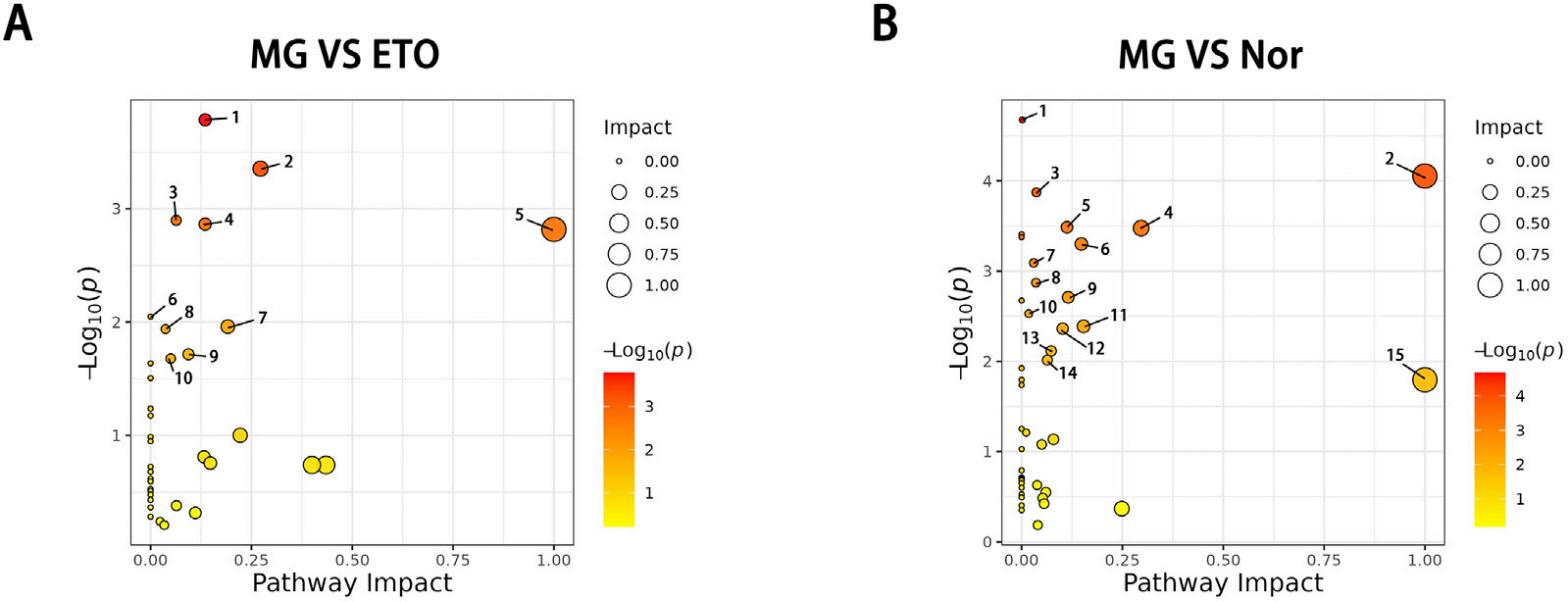
**(A)** The MG vs. ETO groups obtained significantly different metabolic pathways through a combination of metabolite network centering and pathway enrichment. 1, Pyrimidine metabolism. 2, Arachidonic acid metabolism. 3, Nucleotide metabolism. 4, Sphingolipid metabolism. 5, Linoleic acid metabolism. 6, Aminoacyl−tRNA biosynthesis. 7, Glycerophospholipid metabolism. 8, Metabolic pathways. 9, Arginine and proline metabolism. 10, Biosynthesis of amino acids. **(B)** The MG vs. Nor groups obtained significantly different metabolic pathways through a combination of metabolite network centering and pathway enrichment. 1, Biosynthesis of amino acids. 2, Phenylalanine, tyrosine and tryptophan biosynthesis. 3, Metabolic pathways. 4, Phenylalanine metabolism. 5, Citrate cycle (TCA cycle). 6, Arginine biosynthesis. 7, Sphingolipid metabolism. 8, Pyruvate metabolism. 9, Tyrosine metabolism. 10, Valine, leucine and isoleucine degradation. 11, Butanoate metabolism. 12, Glycine, serine and threonine metabolism. 13, Carbon metabolism. 14, Glyoxylate and dicarboxylate metabolism. 15, Linoleic acid metabolism.

The pathway impact map combines the metabolite network center and the pathway enrichment results. As shown in Figures 6A,B linoleic acid metabolism, pyrimidine metabolism, arachidonic acid metabolism, nucleotide metabolism, sphingolipid metabolism, glycerophospholipid metabolism, arginine and proline metabolism, and tryptophan metabolism emerged as crucial metabolic pathways warranting further investigations.

According to the results of KEGG pathway analysis (VIP >1, p < 0.05), Table 1 systematically listed the statistically significant differential metabolites between the ETO intervention group and the model group, among which the characteristic metabolites were screened, 5-methylcytosine, thymine, and hydroxyproline demonstrated the highest variability.

**TABLE 1:** The KEGG pathway enrichment result of differential metabolites between ETO groups.

No.	Metabolite	Formula	KEGG ID	p-value	VIP	Trend Up/Down
1	5-Methylcytosine	C_5_H_7_N_3_O	C02376	0.00012	2.30939	Down
2	Thymine	C_5_H_6_N_2_O_2_	C00178	0.00016	2.24897	Down
3	Hydroxyproline	C_5_H_9_NO_3_	C01157	0.00054	2.15226	Down
4	Phosphocholine	C_5_H_15_NO_4_P+	C00588	0.00054	2.10434	Down
5	Indole-3-acetonitrile	C_10_H_8_N_2_	C02938	0.00057	2.10351	Up
6	Creatinine	C_4_H_7_N_3_O	C00791	0.00173	1.98452	Down
7	Leukotriene B4	C_20_H_32_O_4_	C02165	0.00354	1.90321	Up
8	Arginine	C_6_H_14_N_4_O_2_	C00062	0.00526	1.85293	Down
9	Thymidine	C_10_H_14_N_2_O_5_	C00214	0.00656	1.74740	Down
10	Pseudouridine	C_9_H_12_N_2_O_6_	C02067	0.00901	1.68657	Down
11	Linoleic acid	C_18_H_32_O_2_	C01595	0.01514	1.60228	Up
12	12-OxoETE	C_20_H_30_O_3_	C14807	0.01840	1.60500	Up
13	Sphingosine 1-phosphate	C_18_H_38_NO_5_P	C06124	0.02514	1.47414	Up
14	Sphingomyelin	C_47_H_93_N_2_O_6_P	C00550	0.02627	1.48679	Down
15	Uridine	C_9_H_12_N_2_O_6_	C00299	0.02819	1.43063	Down
16	Citric acid	C_6_H_8_O_7_	C00158	0.03409	1.43169	Down
17	Leucine	C_6_H_13_NO_2_	C00123	0.03851	1.41629	Up
18	6-Hydroxymelatonin	C_13_H_16_N_2_O_3_	C05643	0.04006	1.35825	Up
19	Arachidonic acid	C_20_H_32_O_2_	C00219	0.04560	1.33063	Up
20	Tryptophan	C_11_H_12_N_2_O_2_	C00078	0.04577	1.32326	Up

**FIGURE 7:**
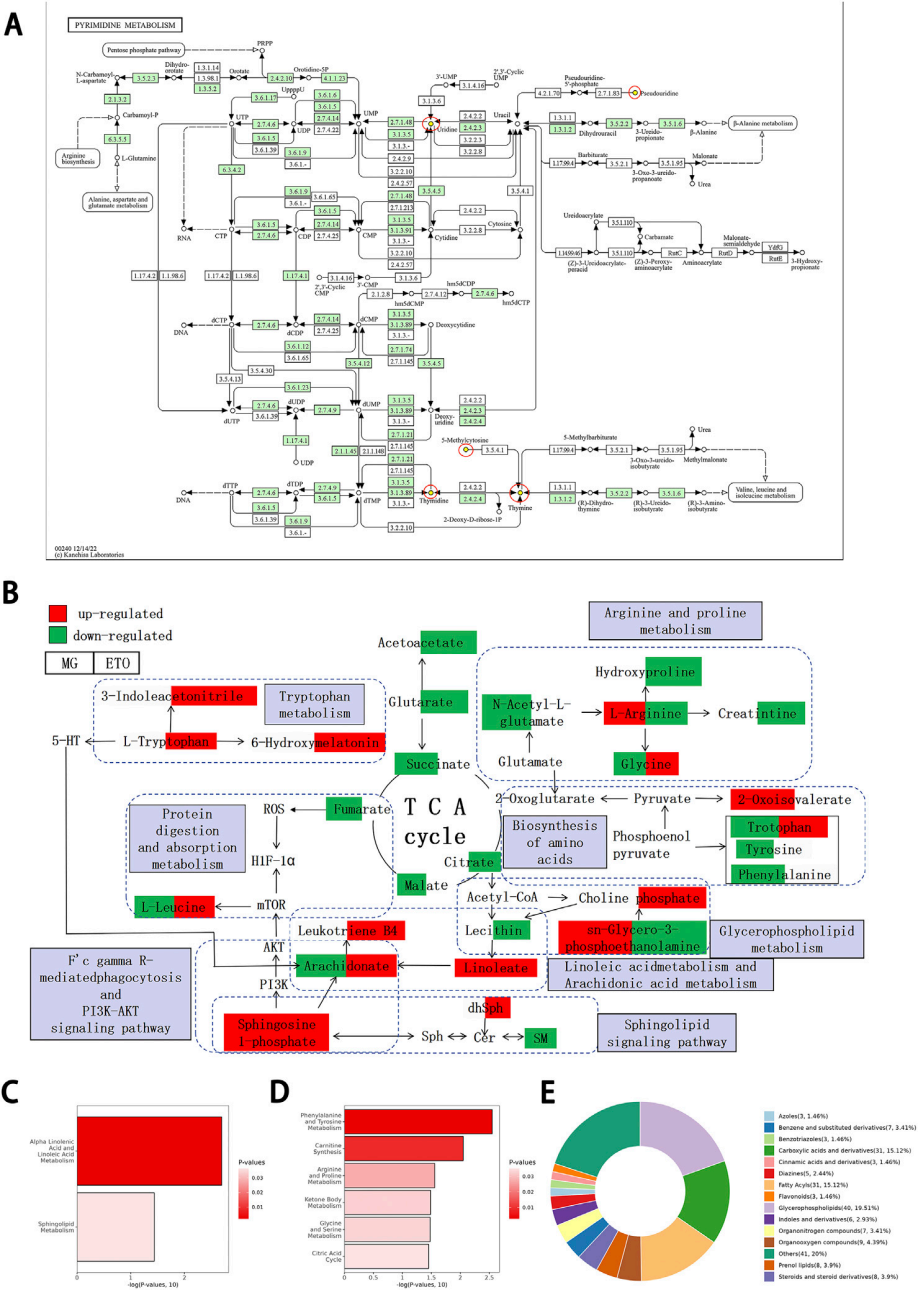
KEGG Enrichment Pathways and Differential Metabolite Classification by Circulation. **(A)** KEGG enrichment pathway of pyrimidine metabolism. Yellow dot indicates downregulated metabolite, arrow indicates reacted direction. Dotted line indicates relationships with other metabolic pathways. Big cycle box represents other metabolic pathways. **(B)** Generalized diagram of the ten metabolic pathways. These include arachidonic acid metabolism, Fc gamma R-mediated phagocytosis, linoleic acid metabolism, glycerophospholipid metabolism, Sphingolipid signaling pathway, biosynthesis of amino acids, protein digestion and absorptionme metabolism, arginine and proline metabolism, tryptophan metabolism, and TCA cycle. Absorptionme metabolism, arginine and proline metabolism, tryptophan metabolism and TCA cycle. **(C)** Enrichment of the metabolic KEGG pathway by ETO in tumor-bearing mice. ETO group compared to MG group. **(D)** Enrichment results of KEGG pathway in MG group compared with Nor group. **(E)** Classification statistics of differential metabolites.

The metabolites uridine, pseudouridine, 5-methyleytosine, thymine, and thymidine were decreased significantly in the pyrimidine metabolic pathway (Figure 7A). Figure 7B presents a generalized diagram of differential metabolic pathways, including differential metabolites encompassing 10 representative interconnected metabolic pathways. The significant differential metabolites screened by KEGG pathway enrichment analysis, and their complete classification information is shown in Supplementary Figures S1, S2. Proteomics studies have confirmed that the therapeutic effect of ETO on tumor-bearing mice is mainly related to arachidonic acid metabolism. Fc gamma R-mediated phagocytosis, linoleic acid metabolism, glycerophospholipid metabolism, sphingolipid signaling pathway, biosynthesis of amino acids, protein digestion and absorption, arginine and proline metabolism, tryptophan metabolism, and TCA cycle.

In protein digestion and absorption metabolism, L-leucine decreased significantly in the model group but increased significantly in the ETO group. In arginine and proline metabolism, L-arginine increased significantly in the model group but decreased significantly in the ETO group. Furthermore, creatinine and hydroxyproline decreased significantly in the ETO group, and N-acetyl-L-glutamate decreased remarkably in the model group. In glycerophospholipid metabolism, choline phosphate showed significant elevation in the ETO group. Both Fc gamma R-mediated phagocytosis and sphingosine 1-phosphate in the sphingolipid signaling pathway were significantly elevated in both model and ETO groups.

Furthermore, ETO treatment promoted a significant increase in AA and its metabolites. Based on the tryptophan metabolic pathway, ETO significantly increased the content of tryptophan and its metabolites 3-indoleacetonitrile and 6-hydroxymelatonin.

The metabolic pathways show intricate interconnections. For instance, L-tryptophan in tryptophan metabolism influences arachidonate through 5-HT, while arachidonate is simultaneously influenced by sphingosine 1-phosphate and linoleate. These interconnected metabolic pathways collectively contribute to tumor growth inhibition in tumor-bearing mice following ETO intervention, as shown by the results.

### 3.6 Major metabolic pathways and mechanism of action of differential metabolites on tumors

The KEGG enrichment analysis identified significant correlations between differential metabolites within two metabolic pathways in the ETO and model groups, indicating their importance in ETO-mediated antitumor effects. These pathways comprised α-linolenic acid and linoleic acid metabolism and the sphingolipid signaling pathway (Figure 7C). The comparison of the model and Nor groups showed enrichment in phenylalanine-tyrosine metabolism, carnitine biosynthesis, and arginine-proline metabolism (Figure 7D). The distinct metabolic profiles between the ETO-treated and untreated groups suggest that ETO exhibits antitumor effects through specific modulation of lipid metabolism. Additionally, ETO-induced metabolic pathway modulation may provide hepatorenal protection by reducing oxidative stress-associated damage. The metabolite categorization loop diagram comparing the ETO vs. model group revealed glycerophospholipids as the most significantly differential component (Figure 7E).

### 3.7 Analysis of the therapeutic effect of ETO on tumor-bearing mice from the perspective of gut microbiota

**FIGURE 8:**
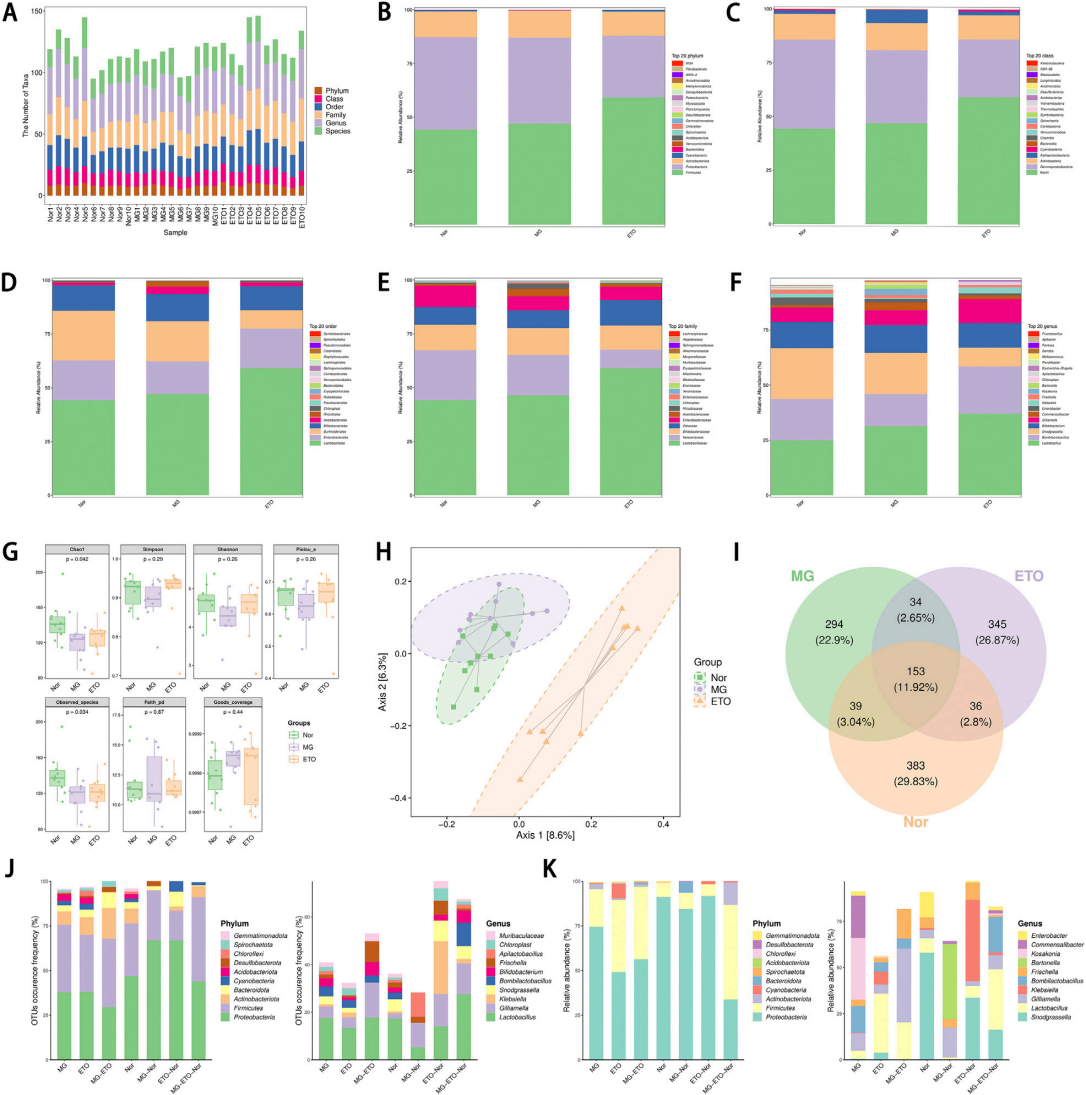
Comparison of changes in the composition of the gut microbiota of the Nor, MG and ETO groups. **(A)** Statistical chart of the number of microbial taxonomic units at each level. **(B–F)** Proportion of different bacteria at phylum, class, order, family, and genus level and comparison between Nor, MG, and ETO groups. **(G)** Grouped box plots of the Alpha Diversity Index. Each panel corresponds to an alpha diversity index, identified in the gray area at the top. In each panel, the horizontal coordinate is the group label, and the vertical coordinate is the value of the corresponding alpha diversity index. In the box-and-line plot, the meanings of the symbols are as follows: upper and lower end lines of the box, upper and lower interquartile range (IQR); median line, median; upper and lower edges, maximum and minimum inner perimeter values (1.5 times the IQR); and points outside the upper and lower edges, indicating outliers. Numbers under the Diversity Index label are p-values for the Kruskal–Wallis test. **(H)** Two-dimensional ordination plot of samples analyzed by PCoA. **(I)** Wayne diagram of sample (group) ASV/OTUs. **(J)** Statistical histogram of the number of ASVs/OTUs in different regions of the Wayne diagram. **(K)** Histogram of ASV/OTU abundance in different regions of the Wayne diagram.

As shown in a recent study, gut microbiota plays a crucial role in modulating tumorigenesis and therapeutic responses (Cheng et al., 2020). By smoothing the sampled data and analyzing the ASV/OTU table, the specific composition of the microbial community in each sample at different taxonomic levels was determined. This analysis enabled the calculation of categorical units within different samples at each classification level. The resulting data were visualized through bar charts using R script to illustrate the number of categorical units at each classification level across different samples (Figure 8A).

Figures 8B–F represent group-averaged species abundance tables that visualize the species composition of each subgroup. These tables were obtained by normalizing abundance values within groups for each species and calculating the average values. At the phylum level, Firmicutes, Proteobacteria, and Actinobacteriota constitute over 90% of total taxonomic sequences. Across the order, phylum, family, genus, and species levels, *Lactobacillus* demonstrated the highest species abundance and most significant inter-group differences, showing a marked increase with ETO treatment.

As illustrated in Figure 8G, the ETO group exhibited reduced bacterial flora abundance and diversity compared to the Nor group. While the model group showed the highest evolutionary-based diversity and the ETO group the lowest, the ETO group demonstrated higher uniformity and coverage than the blank and model groups. These findings suggest that ETO achieves its antitumor and hepatorenal protective effects by reducing mutated flora production, inhibiting harmful bacteria such as Bartonella, and promoting even distribution of gut microbiota throughout the intestinal tract.

**FIGURE 9:**
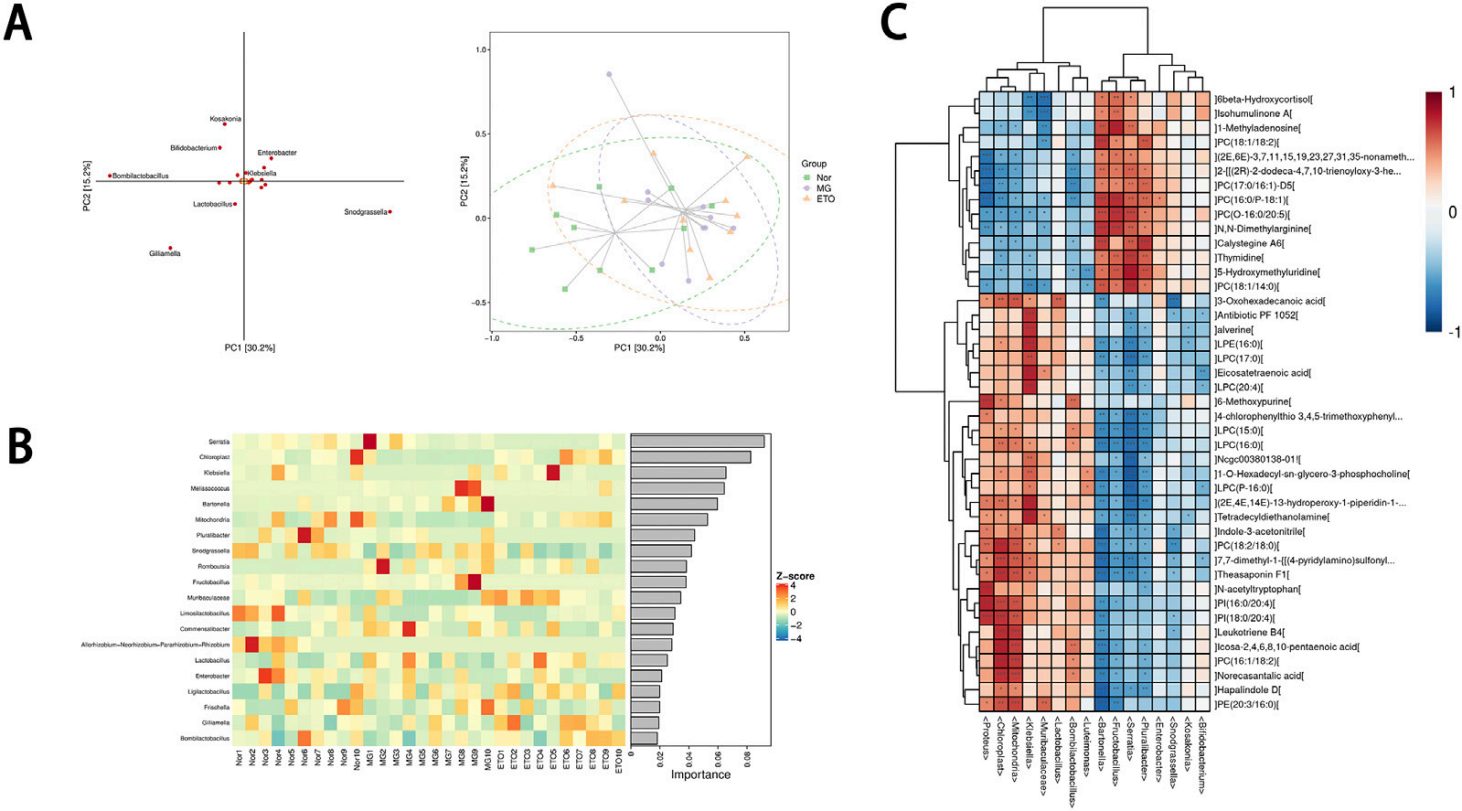
Intergroup and intragroup analysis of intestinal flora, and joint analysis of intestinal flora and metabolomics. **(A)** Species loadings and sample ordering plots for OPLS-DA discriminant analysis. **(B)** Heatmap of the top twenty colonies in terms of genus-level importance. **(C)** Heatmap of correlation hierarchical clustering for the joint analysis of metabolomics and gut flora. Each row represents the number of elements of one type of histology (e.g., Metabonomics) and each column represents the elements of the other type of histology (e.g., Bacteriophage). The left tree branch represents the results of clustering the differential elements of Metabonomics, and the upper tree branch represents the results of clustering analysis of the differential elements of Microbiomics. *** in the small cells of the heatmap denotes a correlation test p-value<0.001, ** denotes a correlation test p-value<0.01, and * denotes a correlation test p. If the input data have elements with classification information and p-value for the difference test, they are also labeled in the figure. Elements clustered to appear in the same cluster have similar correlation patterns.

Significant differences were observed between the ETO group and both model and Nor groups (Figures 8H,I). At the phylum level, Proteobacteria showed the highest number and abundance, while at the genus level, *Lactobacillus* species demonstrated the highest relative number and abundance (Figures 8J,K). Bombilactobacillus contributes relatively significantly to the component differences among sample groups (Figure 9A). Random forest analysis of microbial communities revealed *Serratia*, *Klebsiella*, Bartonella, and *Lactobacillus* as the most important marker species for intergroup differences (Figure 9B).

### 3.8 Nontargeted metabolome and conjoint analysis with the gut flora

To elucidate the relationships between differential metabolites and dominant gut microbiota in the ETO vs. model groups, Spearman’s correlation-based hierarchical clustering analysis was conducted, with results presented through hierarchical clustering heatmaps (Figure 9C). The notable metabolite-microbiota interactions included the following: a negative correlation between the tryptophan metabolite indole-3-acetonitrile and Bartonella; a positive correlation between the tryptophan derivative N-acetyltryptophan and *Proteus*; a positive correlation between *Lactobacillus* abundance and 3-oxohexadecanoic acid; Bombilactobacillus and Fructobacillus were positively correlated with 6-methoxypurine and N,N-dimethylarginine (a key metabolite in arginine-proline metabolism), respectively; *Serratia* abundance was positively correlated with the pyrimidine metabolites 5-hydroxymethyluridine and thymidine. These findings suggest that ETO modulates host-microbiota metabolism to achieve therapeutic effects.

## 4 Discussion

HCC is one of the most prevalent malignant tumors and ranks as the third leading cause of cancer-related death globally (Bray et al., 2024). Numerous compounds derived from edible and medicinal mushroom sources have exhibited multiple pharmacological effects (Zhao et al., 2020; Li and Bao, 2022). This study evaluated the antitumor effect of ETO extracted from P. adiposa. The results revealed that the intraperitoneal administration of ETO effectively delayed transplanted tumor growth in H22 tumor-bearing mice with minimal toxicity through modulation of metabolic pathways and gut microbiota, thus indicating the potential of ETO as a therapeutic candidate for tumors.

ETO intervention demonstrated significant tumor inhibitory effects on H22 tumor-bearing mice. The ETO group showed no significant adverse effects in Western blotting assay, H&E staining, TUNEL assay, and ELISA analyses. These findings indicated that ETO effectively inhibited tumor growth in H22 tumor-bearing mice without substantial toxicity.

In addition, based on the results of Western blotting assay, H&E staining, TUNEL assay, and ELISA, and analyzing the potential toxic effects of ETO, it can be learned that the damage of ETO to organs such as liver and kidney is less than that of CTX, which proves that its safety is higher than that of CTX and can be presumed to be suitable for clinical application.

The analysis of metabolic pathways and metabolites revealed that the antitumor effects of ETO correlate strongly with several key pathways: pyrimidine metabolism, arachidonic acid metabolism, FcγR-mediated phagocytosis, linoleic acid metabolism, glycerophospholipid metabolism, sphingolipid signaling pathway, amino acid biosynthesis, protein digestion and absorption metabolism, arginine and proline metabolism, tryptophan metabolism, and TCA cycle. Notably, all these pathways, except pyrimidine metabolism, are linked to the TCA cycle.

Tryptophan and its metabolites demonstrate antioxidant properties, influence immune responses, and function as anabolic signals. Indoles exhibit anticancer and antitumor properties (Ahmad et al., 2010; Shashi et al., 2023; Yao et al., 2024). L-arginine in arginine and proline metabolism induces vasodilation and enhances blood flow (Zhang et al., 2001; Gökçe, 2004; Al-Koussa et al., 2020). Increased vascular perfusion expands the tumor-vascular interface, potentially facilitating nutrient uptake and tumor proliferation. This explains the elevated L-arginine levels observed in the model group, while its suppression in the ETO group supports the antitumor efficacy of ETO.

Sphingosine 1-phosphate in FcγR-mediated phagocytosis and the sphingolipid signaling pathway modulates lymphocyte trafficking, vascular permeability, and inflammatory responses through S1PR1 binding (Pyne and Pyne, 2010; Pyne and Pyne, 2020; Zou et al., 2023). Figure 6B shows elevated S1P in both model and ETO groups, indicating immune activation against xenografts. The interaction between S1P and S1PR1 enhances lymphocyte migration to inflammatory sites, with ETO amplifying this response to inhibit tumor growth. Additionally, S1P upregulation in ETO-treated mice enhances arachidonic acid production.

Alpha-linolenic acid metabolizes DHA, EPA, and DPA, which reduce cholesterol and triglyceride levels while promoting saturated fatty acid metabolism, thereby decreasing blood viscosity, enhancing circulation, and improving tissue oxygenation to reduce fatigue (Zhao et al., 2007; Erdinest et al., 2012). These findings suggest that ETO functions as an antitumor and hepatorenal protective agent by regulating this metabolic pathway (Burdge, 2006; Yuan et al., 2022). Linoleic acid primarily affects the skin, cell membrane, and arachidonoid-related processes (Ip et al., 1999). When mice experience significant heat loss through the skin, normal growth is inhibited, and linoleic acid restores excessive surface water loss, thereby reducing growth impairment (Kinsella, 1991). These results demonstrate the significant role of ETO in maintaining normal physiological health.

Sphingolipid metabolism plays a crucial role in regulating cell growth, differentiation, and programmed cell death (Janneh and Ogretmen, 2022; Li et al., 2022). The current understanding of their function centers on the complex interaction of sphingolipids, such as gangliosides, with growth factor receptors, extracellular matrix, and adjacent cells (Spiegel and Merrill, 1996). Sphingolipids constitute essential building blocks of nucleated cell membranes, and their metabolic intermediates participate in transducing extracellular signals to the cell interior. Ceramides can be generated from sphingolipids or through *de novo* synthesis in response to vitamin D3, tumor necrosis factor alpha, gamma-interferon, or interleukin 1 (Kolter and Sandhoff, 2006; Gaggini et al., 2022). In most cell types, ceramides mediate antiproliferative responses, including cell differentiation, cell cycle arrest, cellular senescence, or apoptosis (Ruvolo, 2003). Evidence suggests that ETOs likely inhibit tumor cell growth by affecting the tumor cell membrane. Glycerophospholipids exhibit significant differences, serving as crucial components of the cell membrane by providing stability, fluidity, and permeability. These compounds are essential for the proper functioning of membrane protein receptors and ion channels (Hishikawa et al., 2014). Thus, ETO may act as an antagonist against tumor cells by specifically affecting the physiological functions of the cell membrane (Szlasa et al., 2020).

By comparing the gut microbiota of the ETO, Nor, and model groups, we found that *Lactobacillus* had the highest species abundance in the ETO group, and that Firmicutes and *Lactobacillus* showed increased in abundance in the ETO group; both Firmicutes and *Lactobacillus* as beneficial bacteria (Shi et al., 2020; Cheng and Liu, 2020; Hashmi et al., 2020; Slover and Danziger, 2008). This indicates that ETO affects the composition and stability of the gut microbiota. ETO suppressed pathogenic bacterial evolution while enhancing intestinal colonization by the beneficial flora (e.g., *Lactobacillus*), thereby restoring gut microbiota homeostasis. The integrated pharmacomicrobiomics analysis revealed systemic metabolic reprogramming through blood circulation as a key mechanism: ETO first altered plasma metabolites (particularly TCA cycle intermediates and sphingolipids), creating a tumor-suppressive microenvironment. This metabolic shift subsequently modulated microbial composition through enterohepatic circulation, amplifying antitumor efficacy while mitigating hepatorenal toxicity through dual metabolic-microbial regulation.

The metabolite L-leucine in protein digestion and absorption metabolism can significantly promote axon growth and regeneration (Ma et al., 2021). Its significant decrease in the model group but significant increase in the ETO group suggests that ETO may affect axon regeneration and nerve function restoration. Additionally, the tryptophan metabolic pathway and its metabolites, such as 6-hydroxymelatonin, act as regulators of circadian rhythms and can therefore modulate sleep disorders or cognitive functions (Kanova and Kohout, 2021). This finding suggests that ETO may also play a role in regulating the nervous system.

## 5 Conclusion

The present study demonstrated that ETO significantly inhibited the growth of HCC transplantation tumors and thus could be a candidate for treating HCC. The antitumor mechanism of ETO primarily involves inhibition of tumor growth through modulation of lipid and steroid metabolism, complemented by a significant increase in metabolites such as choline phosphate, AA, and tryptophan, which contribute to tumor growth suppression. The antitumor effects of ETO are closely associated with metabolites such as glycerophospholipids and beneficial bacteria including lactobacilli. While further investigation is necessary to fully elucidate the anti-HCC tumor mechanism of ETO, this study provides a foundation for the subsequent development and clinical application of ETO.

## Data Availability

The data presented in the study are deposited in the NCBI repository, accession number PRJNA1375352. Available at: https://www.ncbi.nlm.nih.gov/sra/PRJNA1375352.
