# Supplementary figures and images for: Integrated gut microbiota and metabolomics analysis reveals the antitumor effects of ergosta-4,6,8(14),22-tetraen-3-one purified from the medicinal fungus pholiota adiposa in tumor-bearing mice
Source: Front Pharmacol. 2025 Dec 16;16:1653035. doi: 10.3389/fphar.2025.1653035 (PMC12748264; doi:10.3389/fphar.2025.1653035)

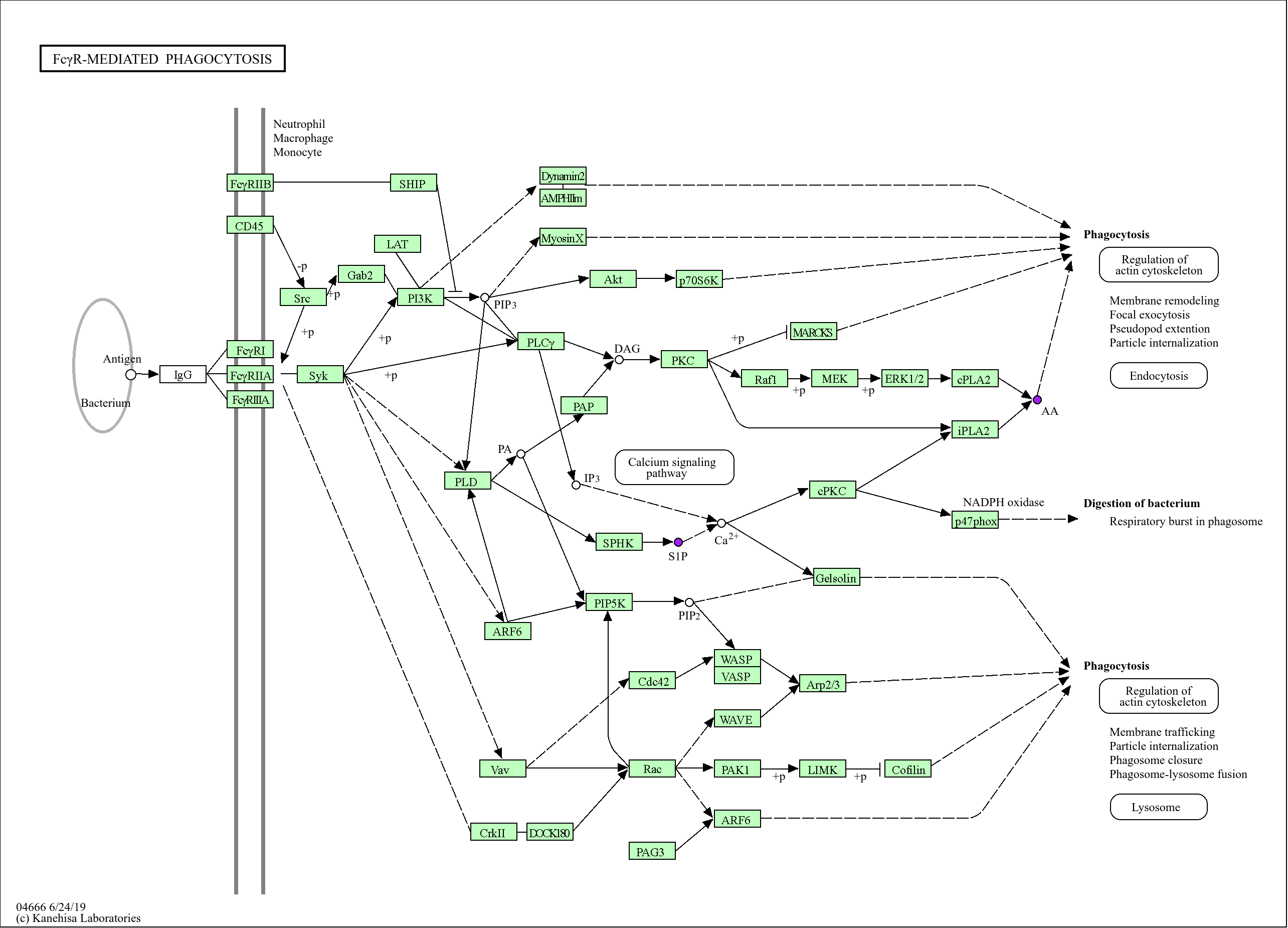

Supplement: Supplementary file 1 [file Image14.png]

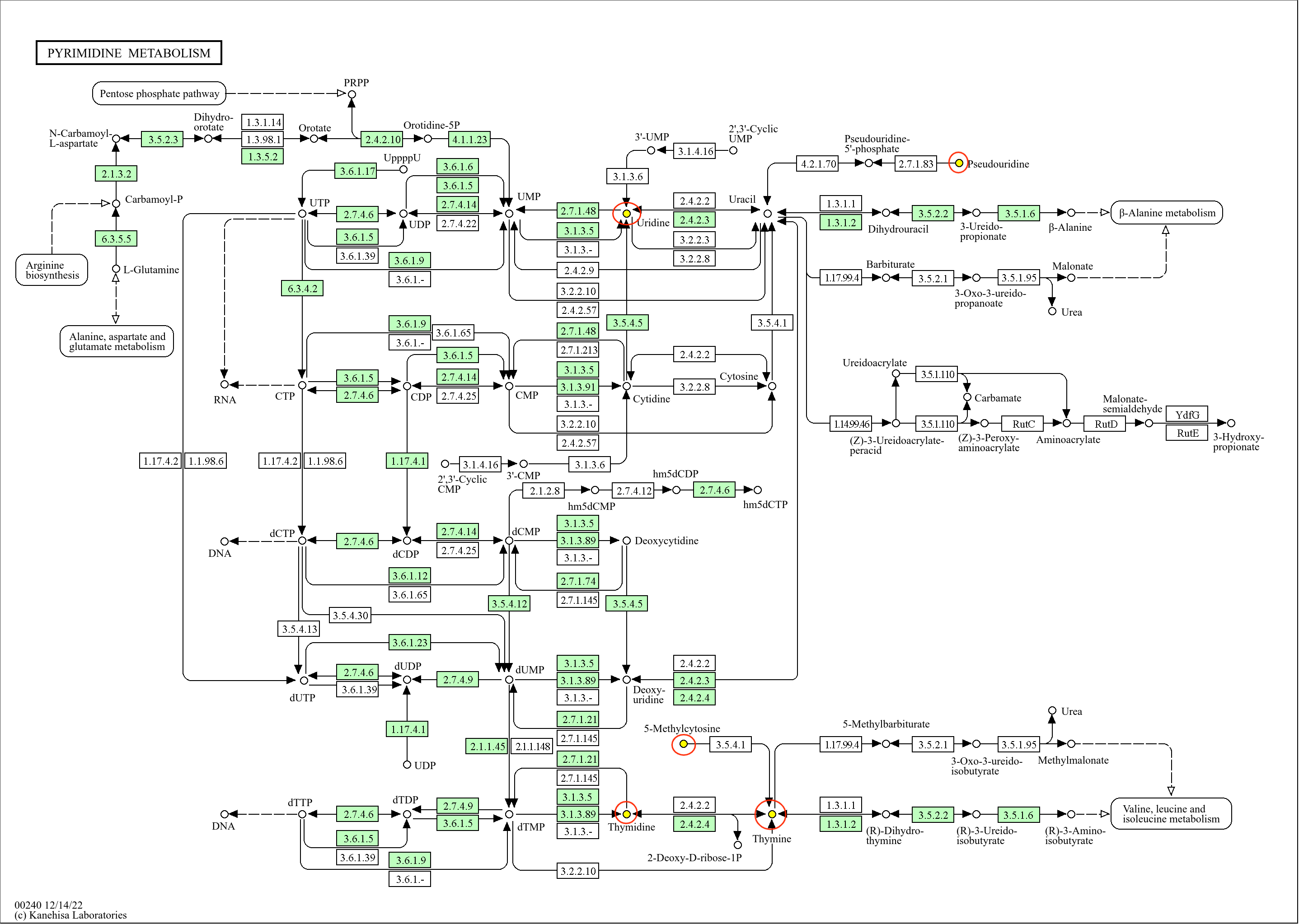

Supplement: Supplementary file 2 [file Image11.png]

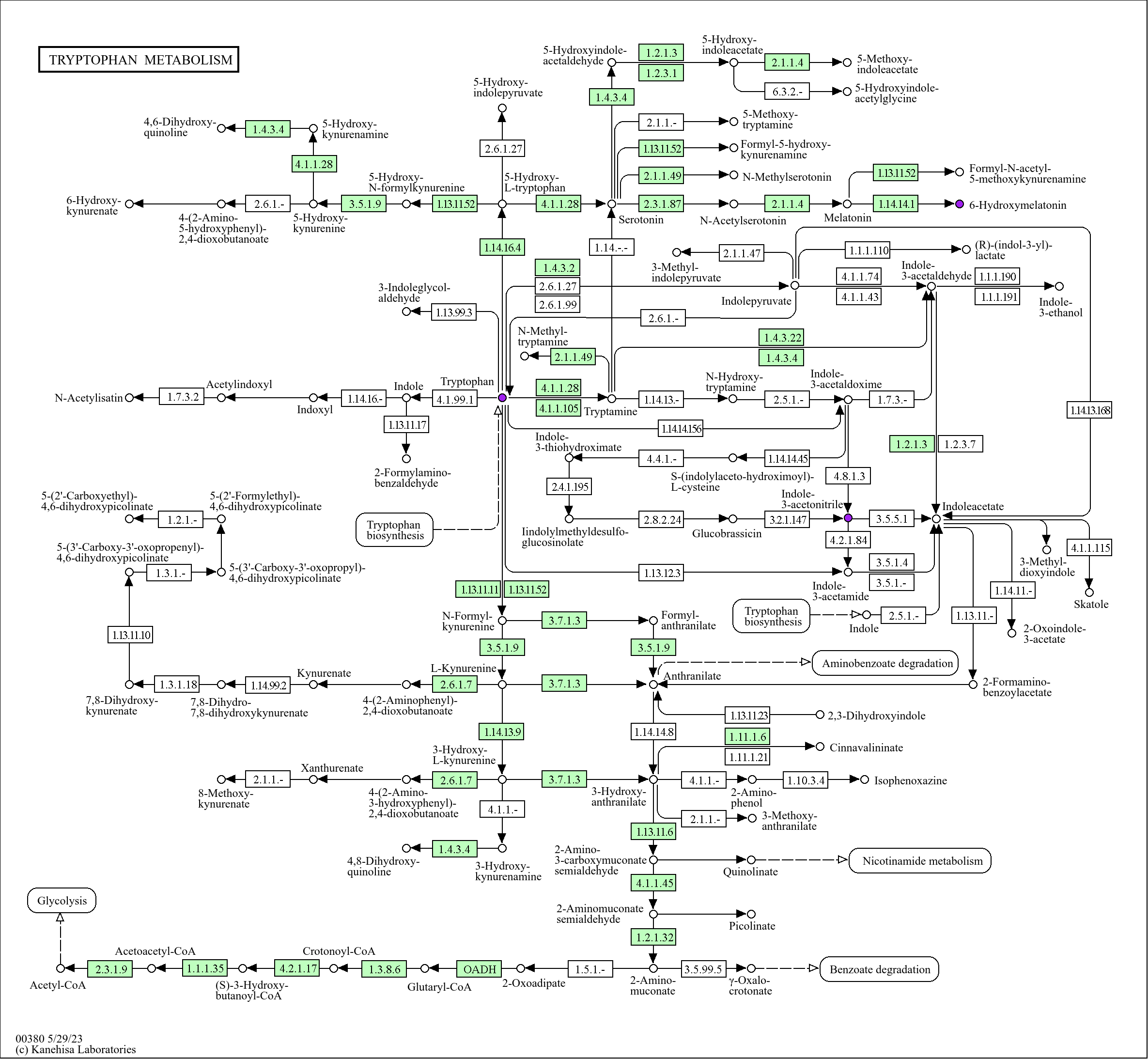

Supplement: Supplementary file 4 [file Image12.png]

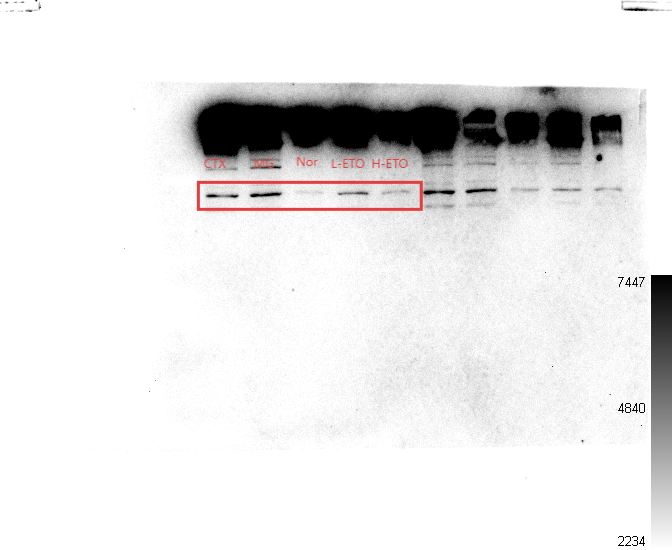

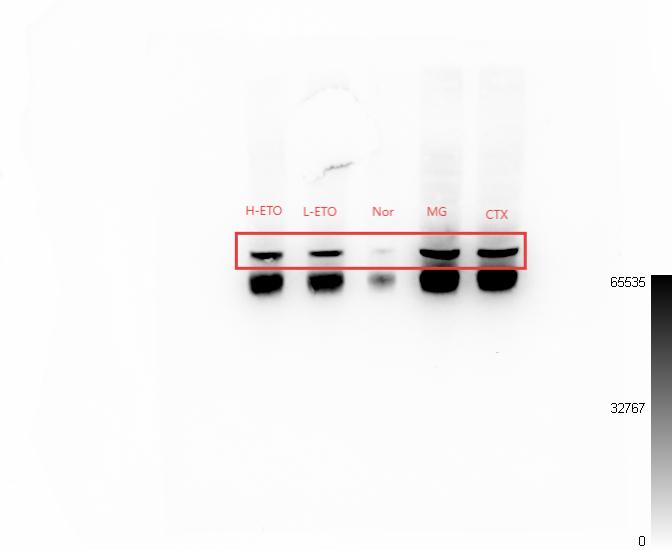

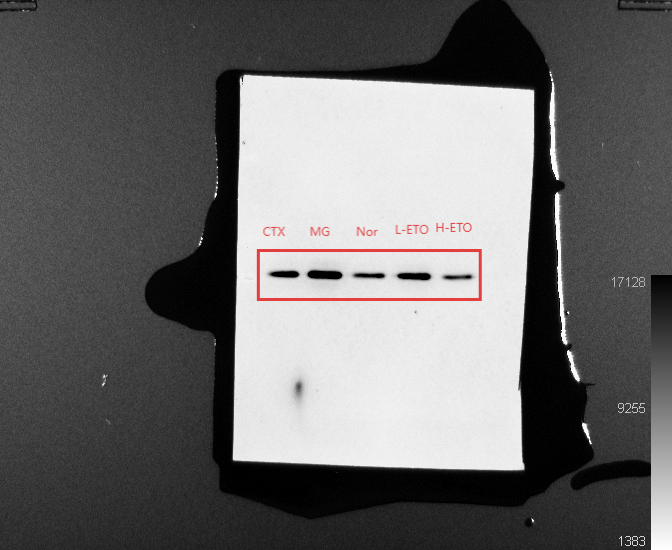

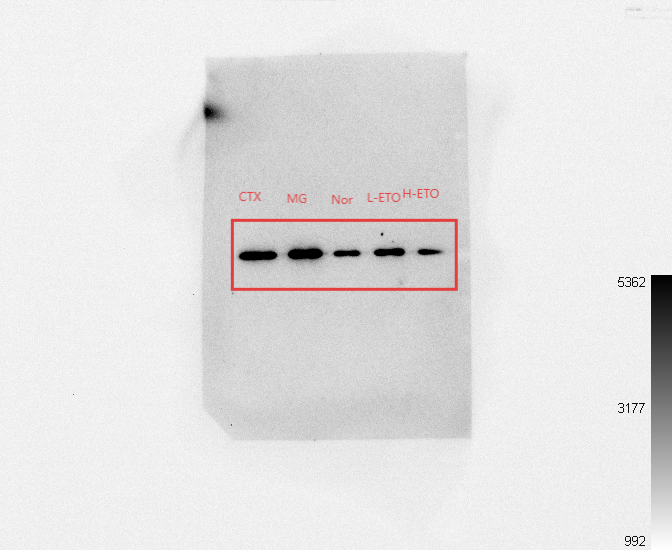

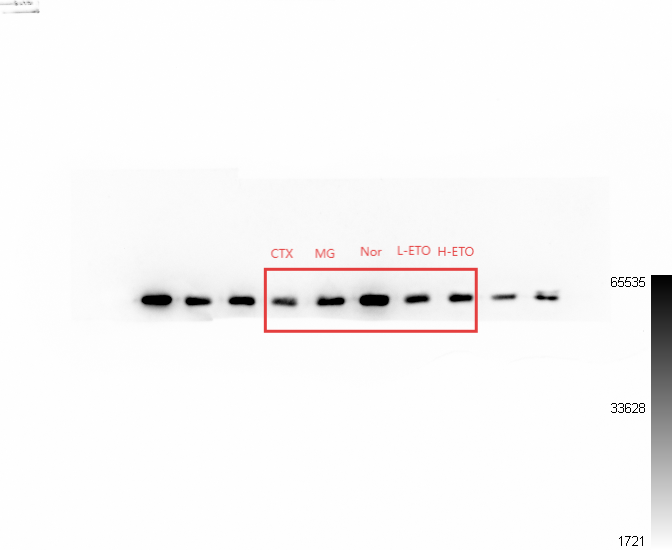

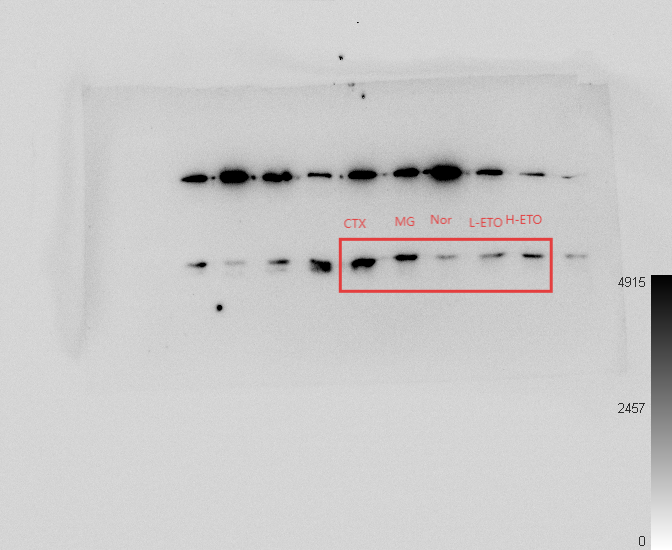


NF-kB

IKB

P-IKB


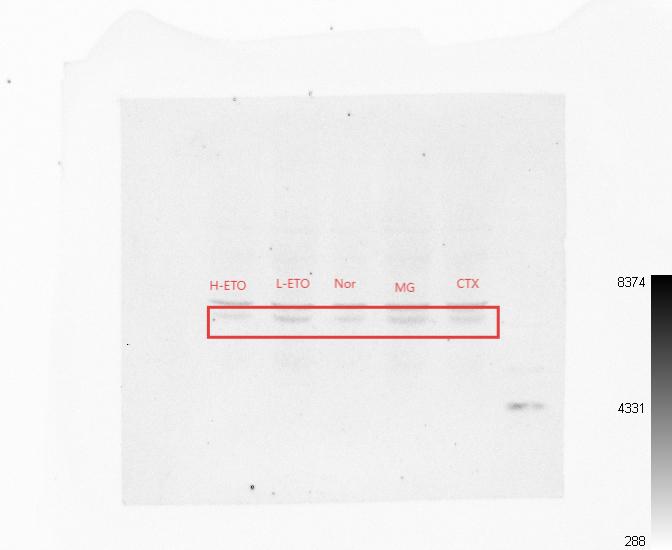


IKB

c-caspase-3

caspase-3

BAX

BCL-2

Supplement: Supplementary file 5 [file Supplementaryfile3.docx]

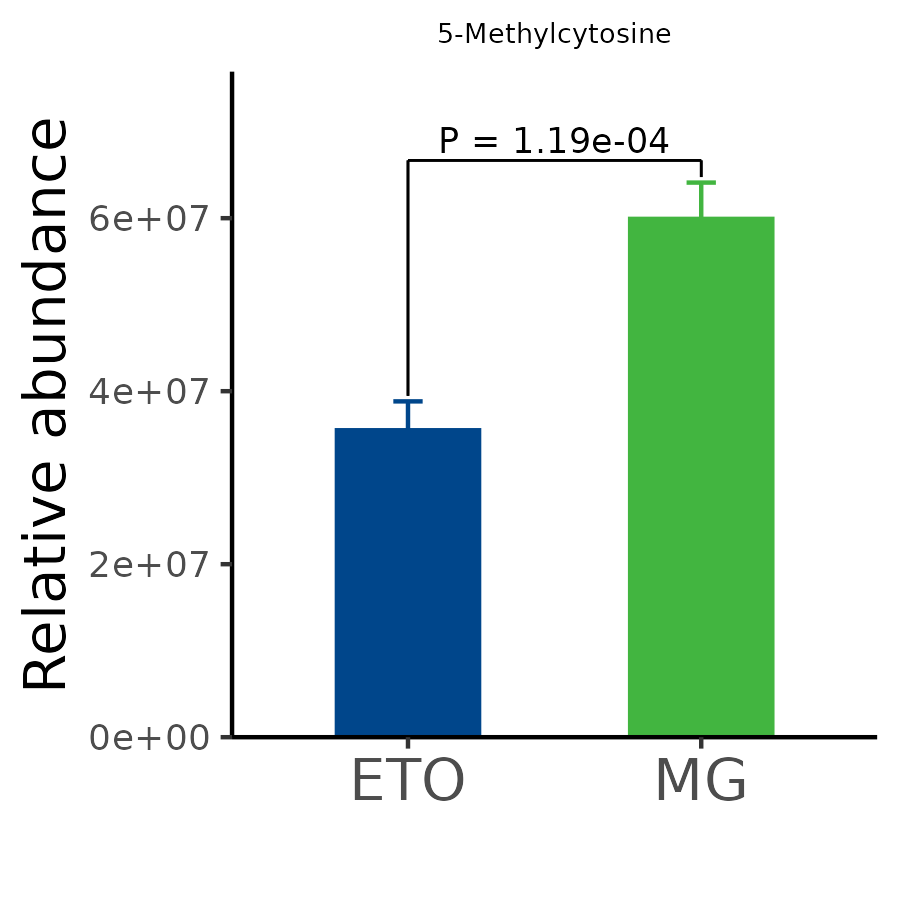

Supplement: Supplementary file 6 [file Image5.png]

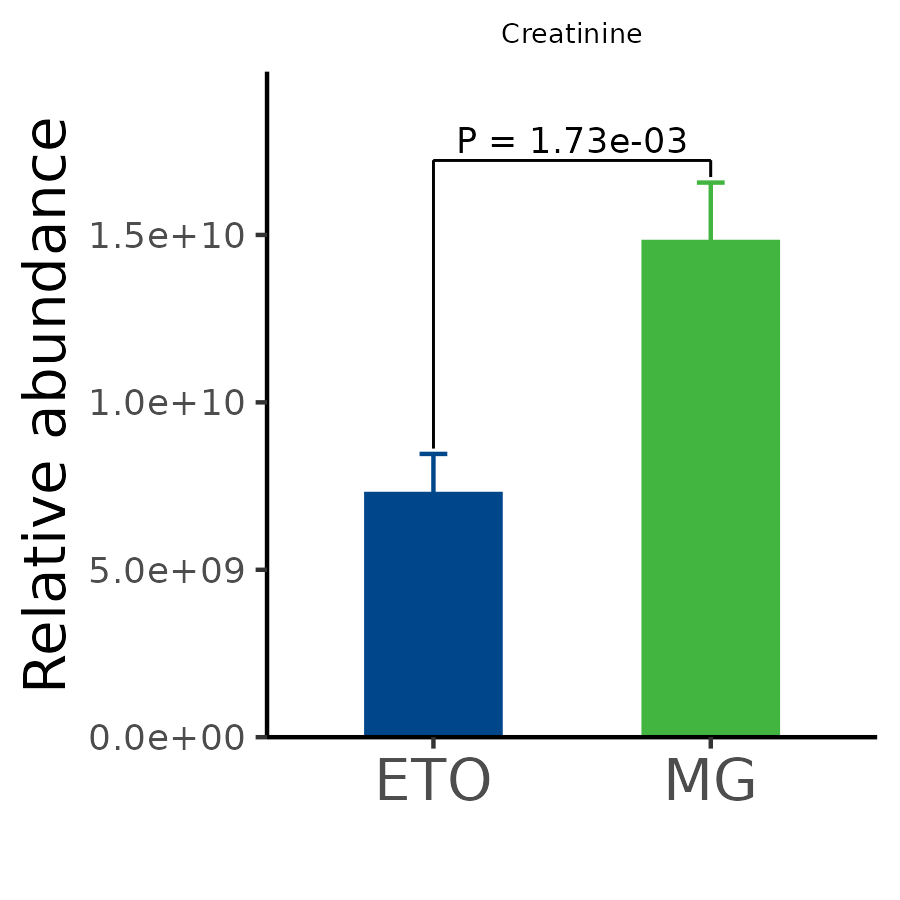

Supplement: Supplementary file 7 [file Image4.png]

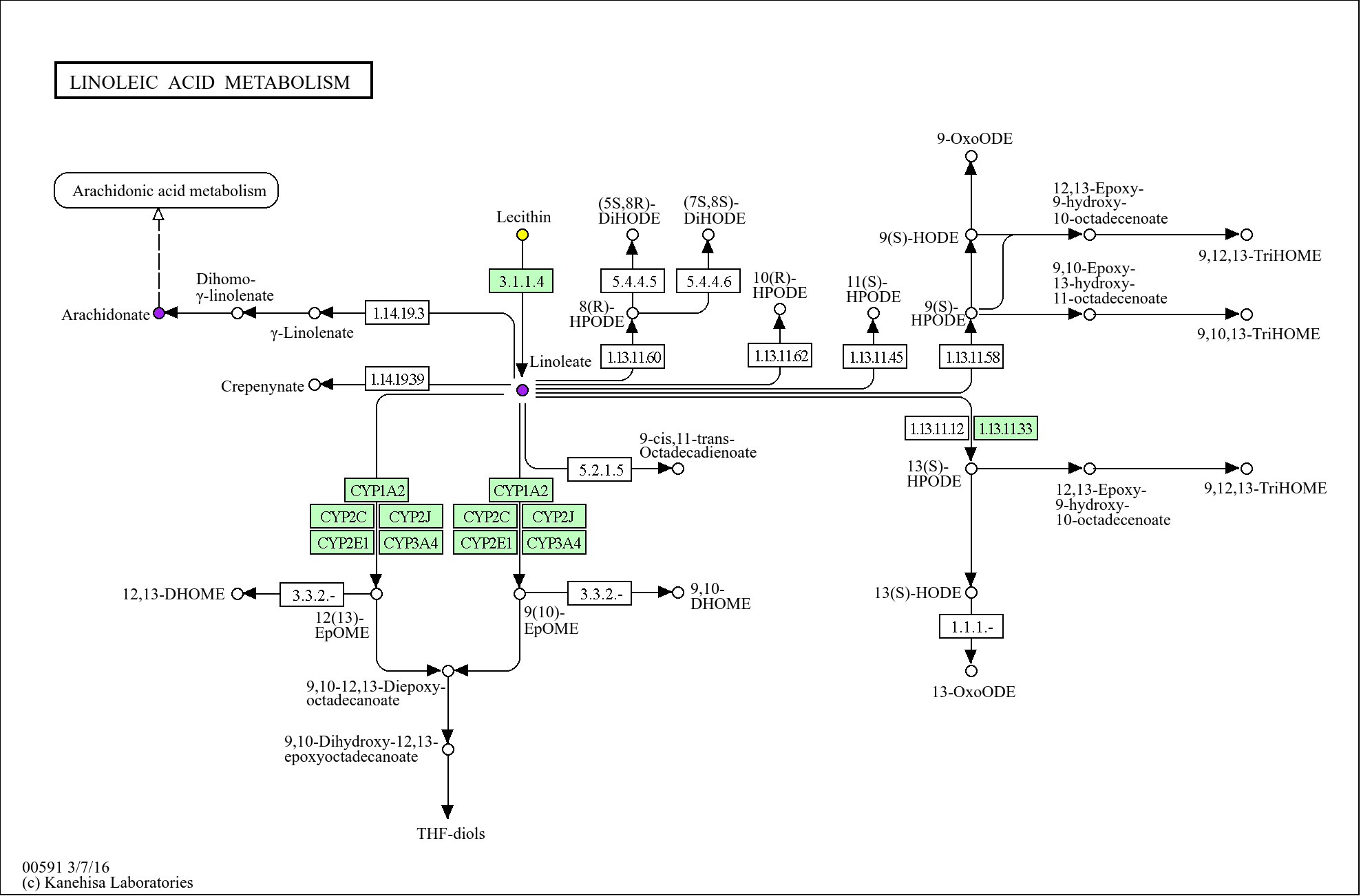

Supplement: Supplementary file 8 [file Image13.png]

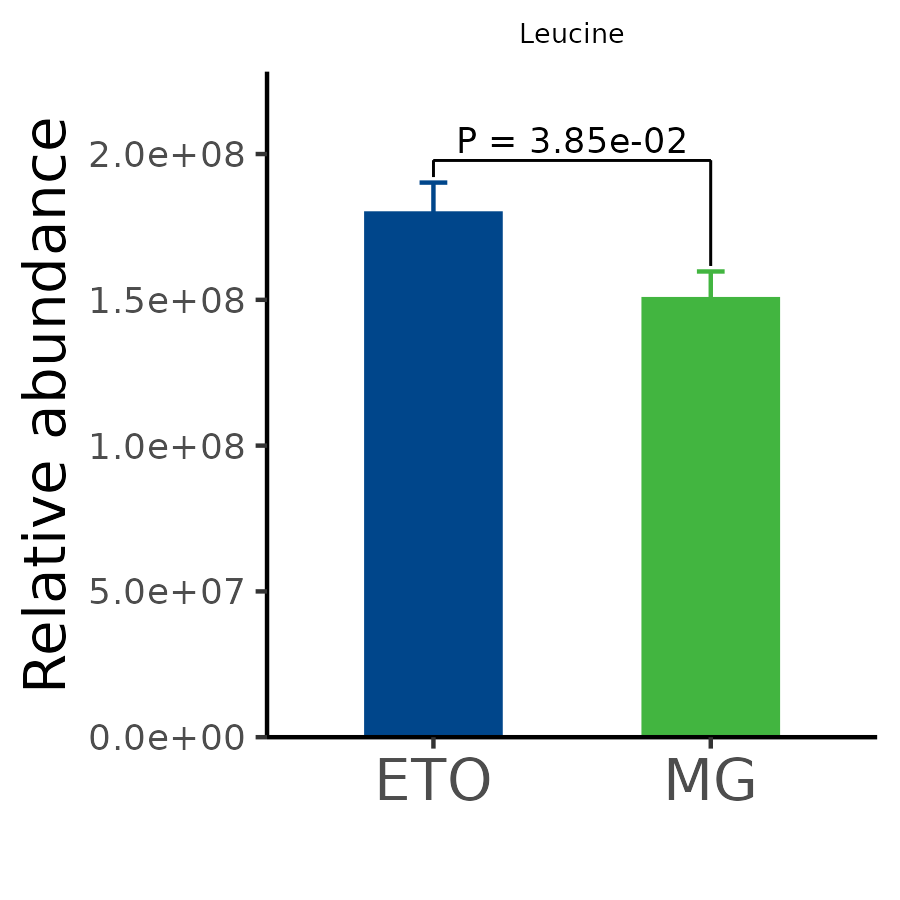

Supplement: Supplementary file 9 [file Image7.png]

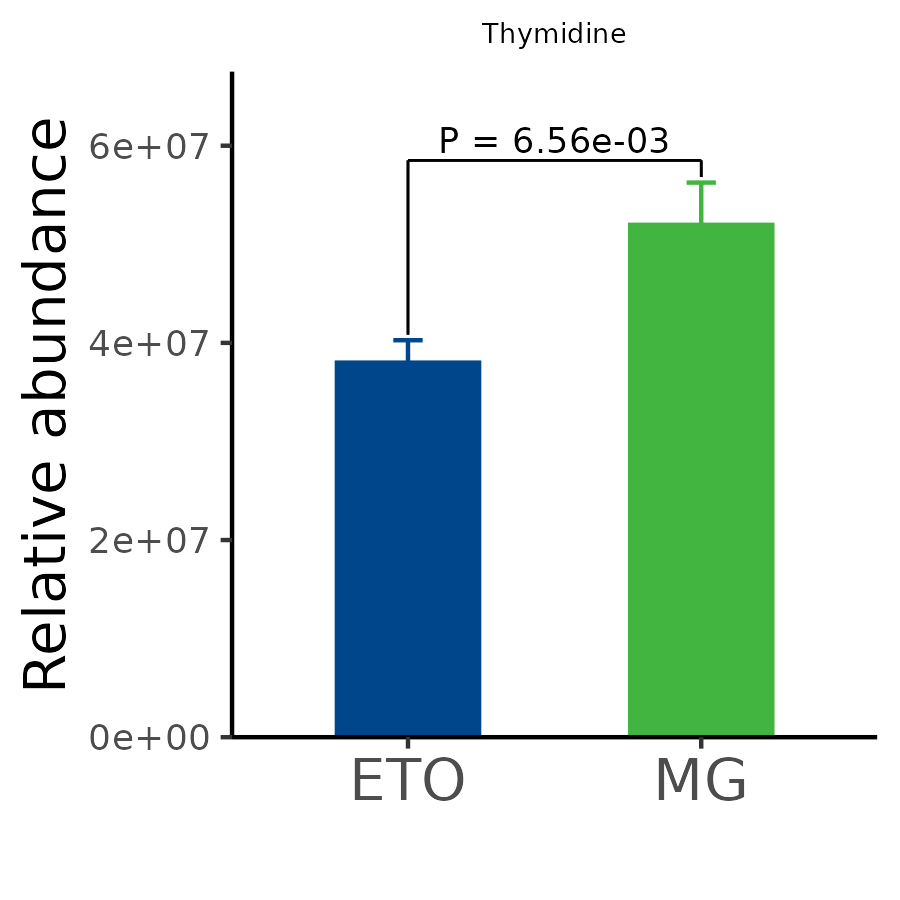

Supplement: Supplementary file 10 [file Image2.png]

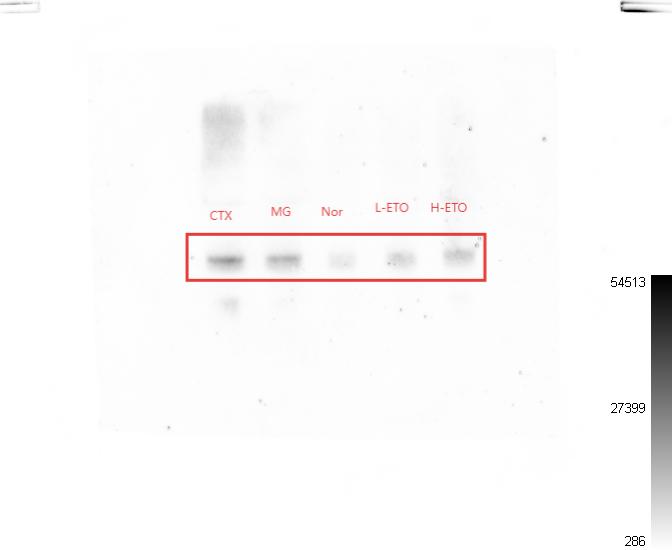

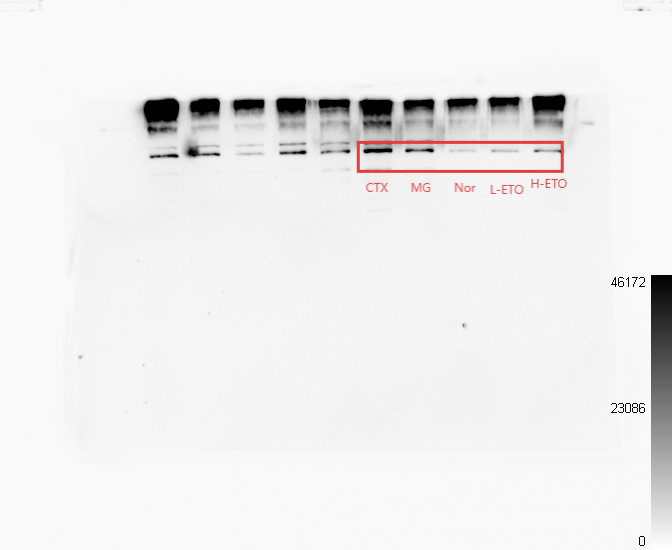

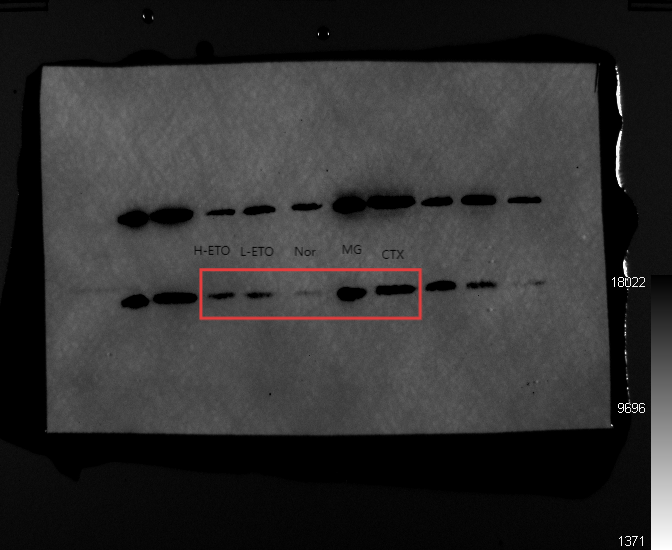

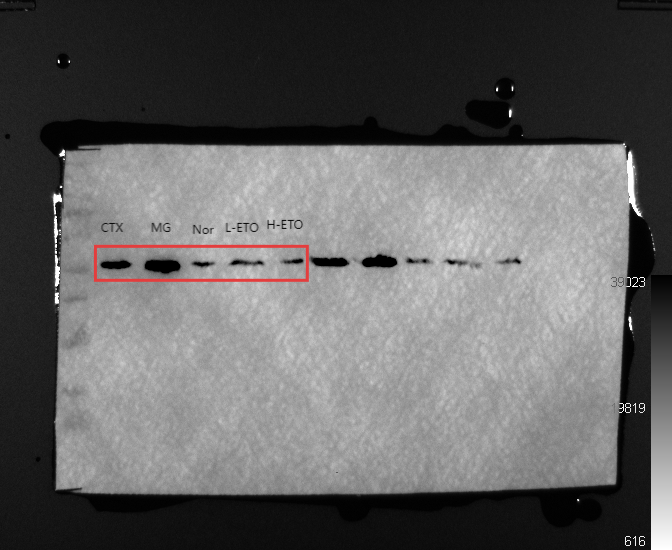

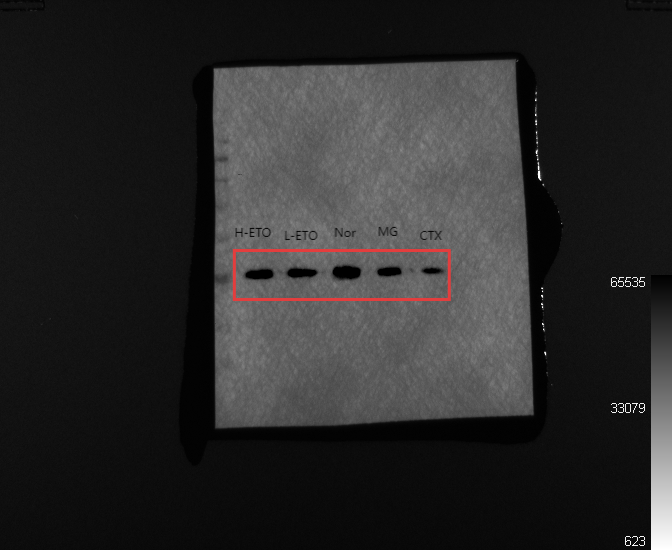

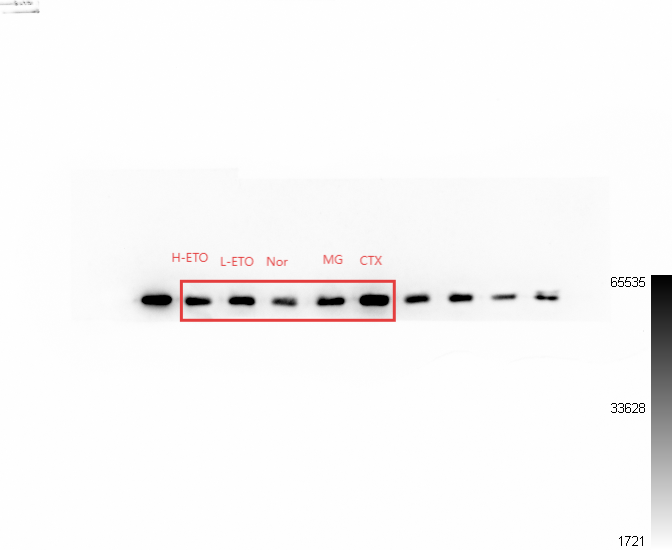


NF-kB

P-IKB

Caspase-3

IKB

BAX

BCL-2

Supplement: Supplementary file 11 [file Supplementaryfile2.docx]

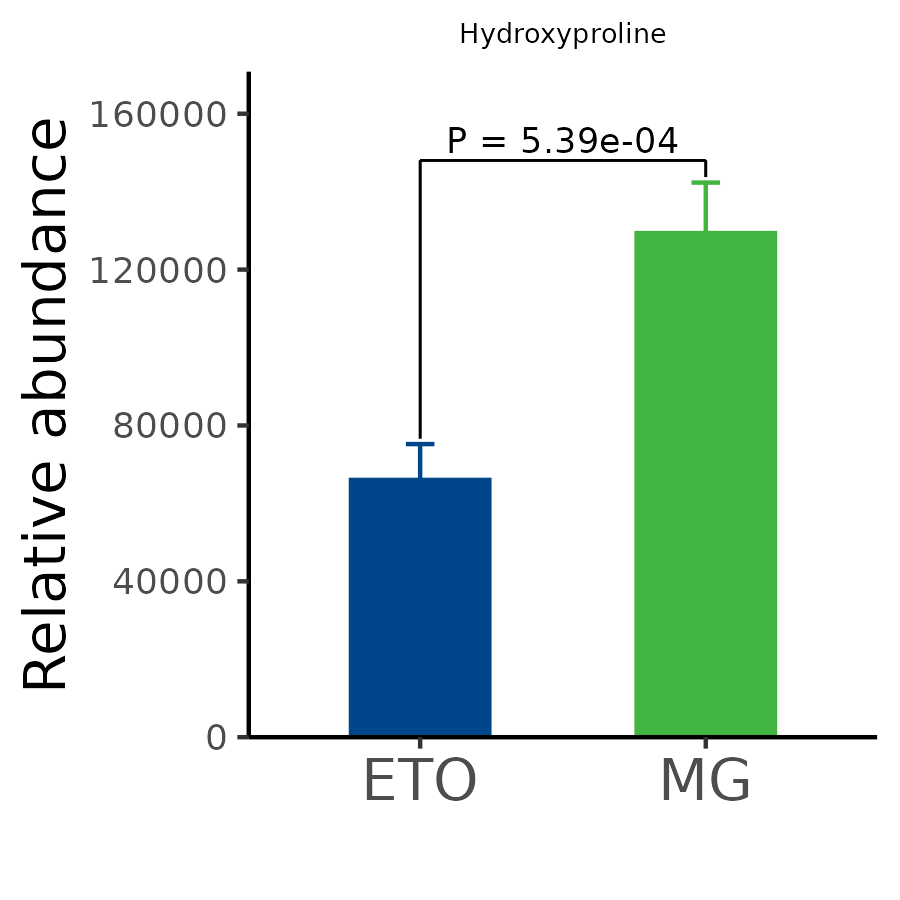

Supplement: Supplementary file 12 [file Image1.png]

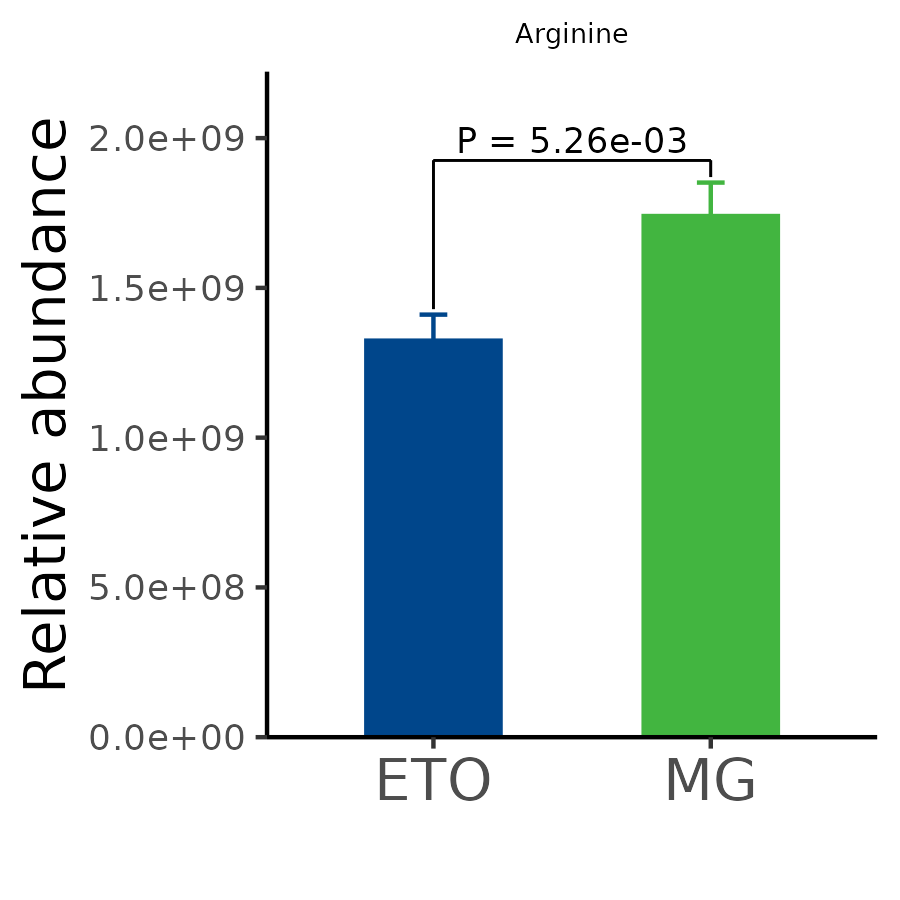

Supplement: Supplementary file 13 [file Image8.png]

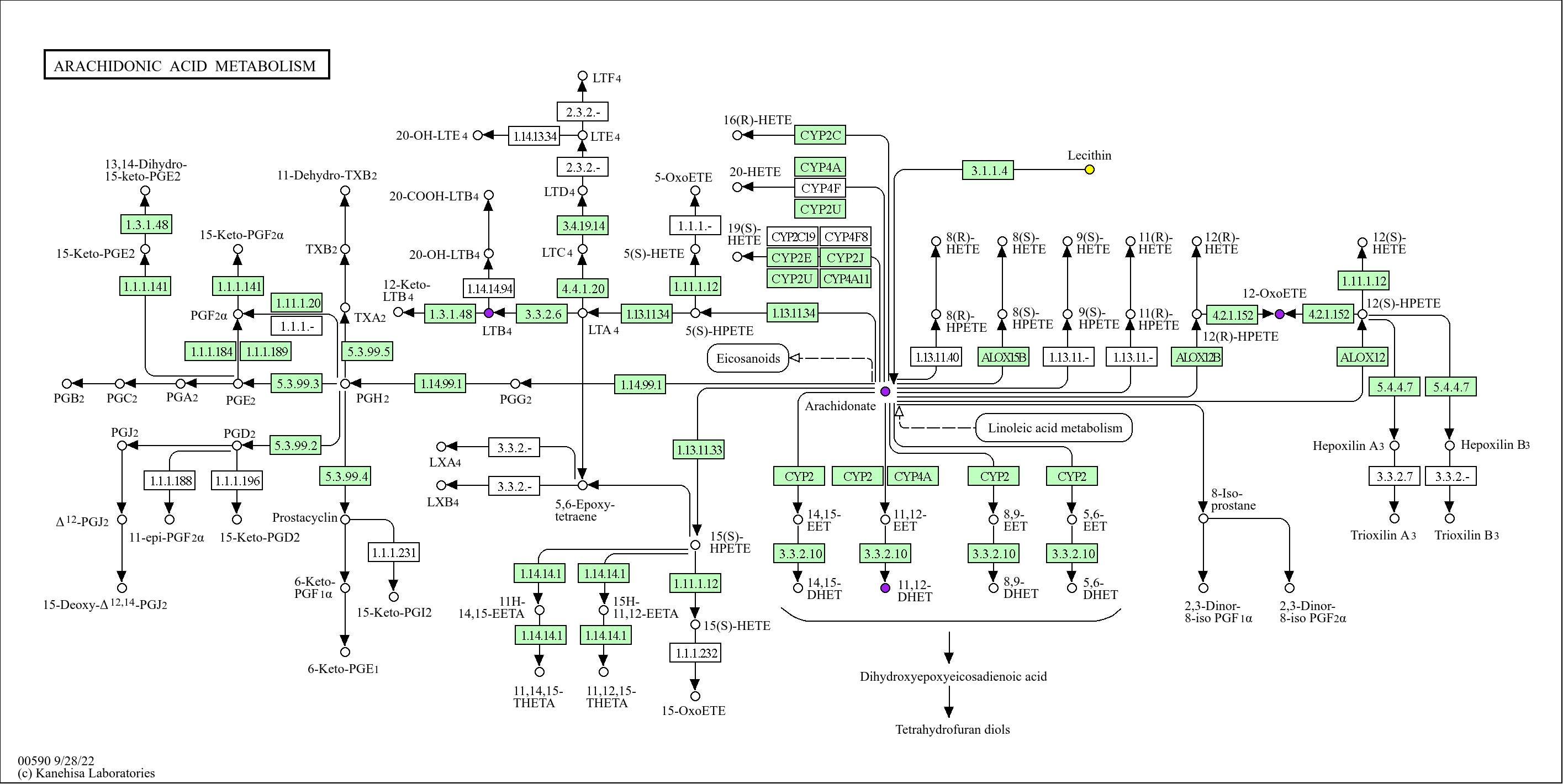

Supplement: Supplementary file 14 [file Image9.png]

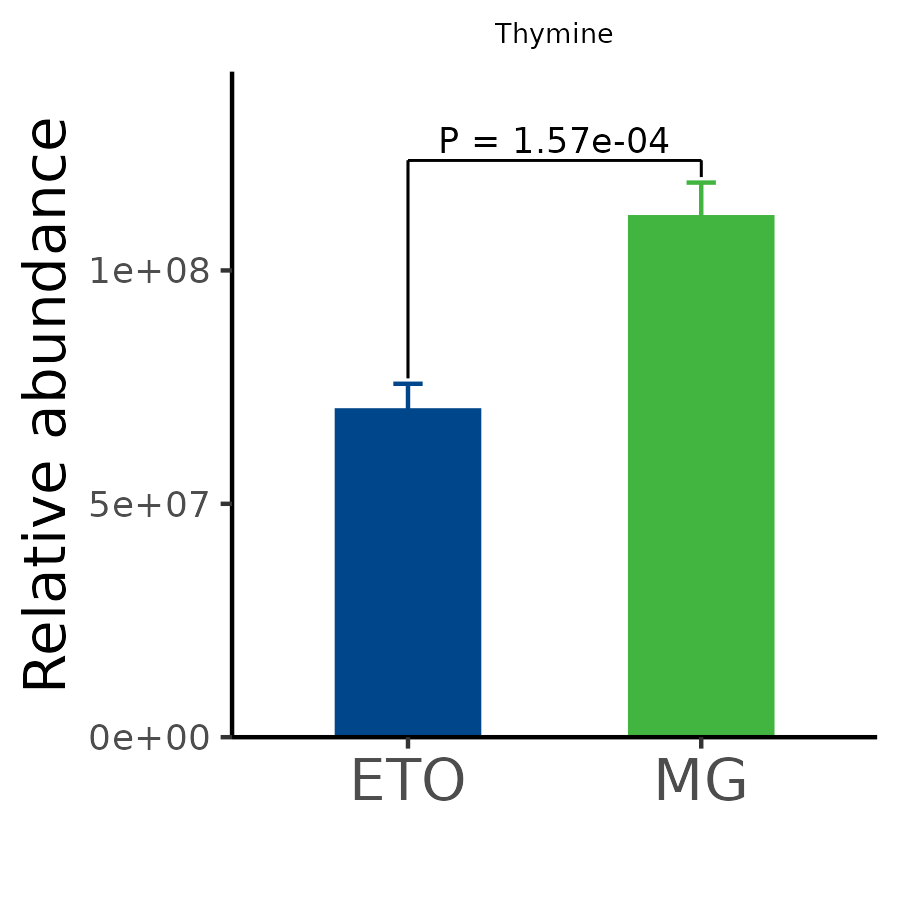

Supplement: Supplementary file 15 [file Image6.png]

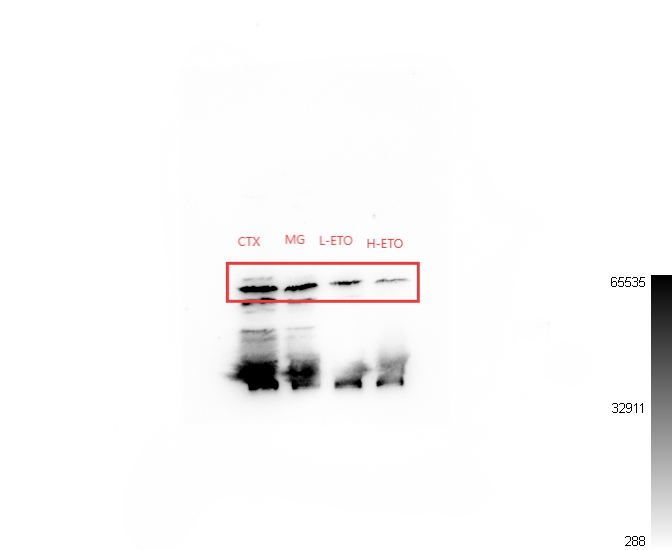

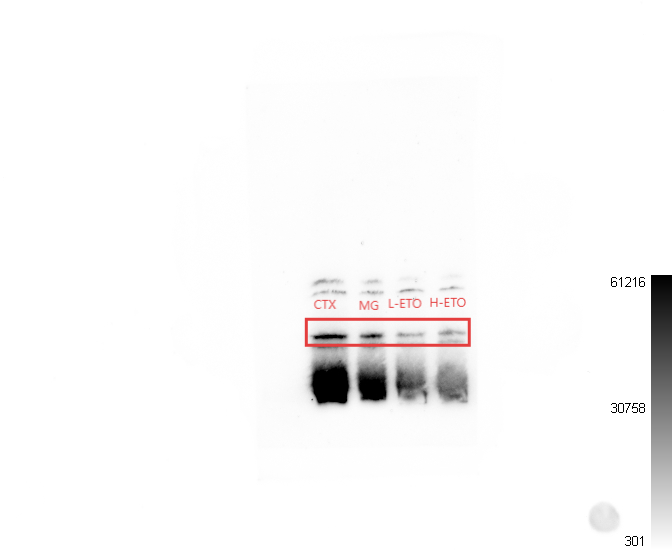

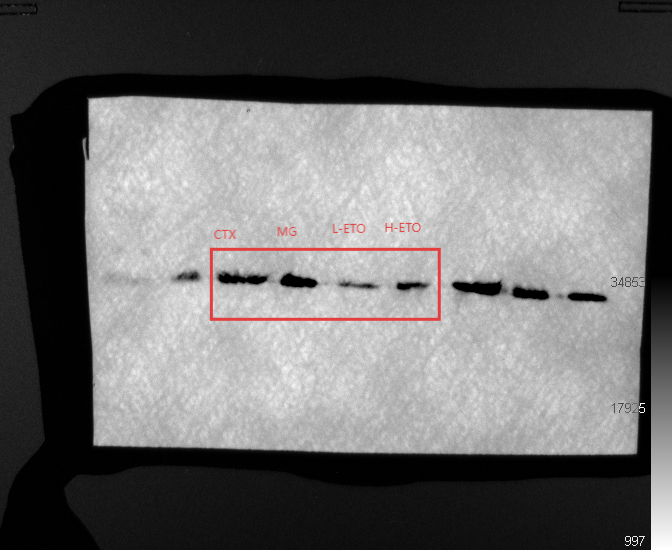

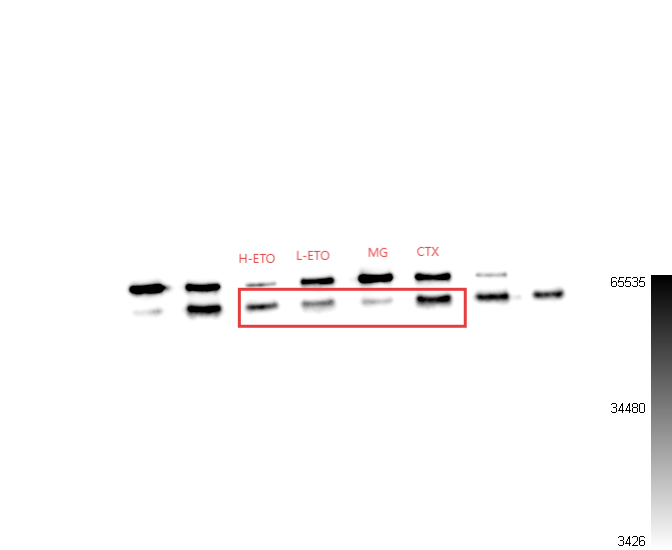

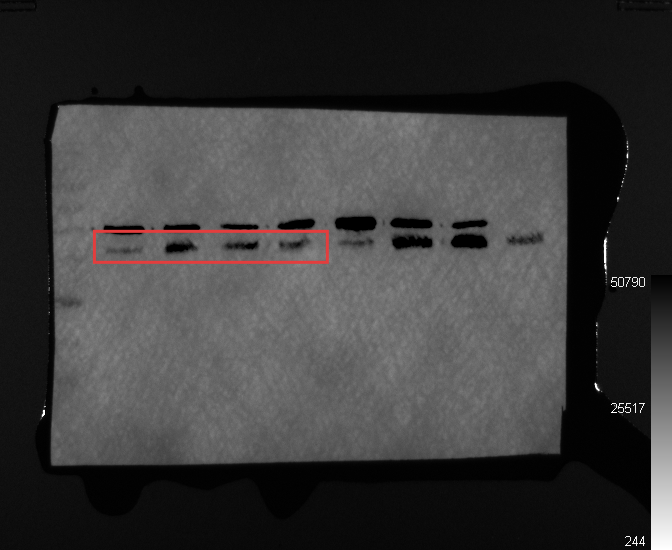

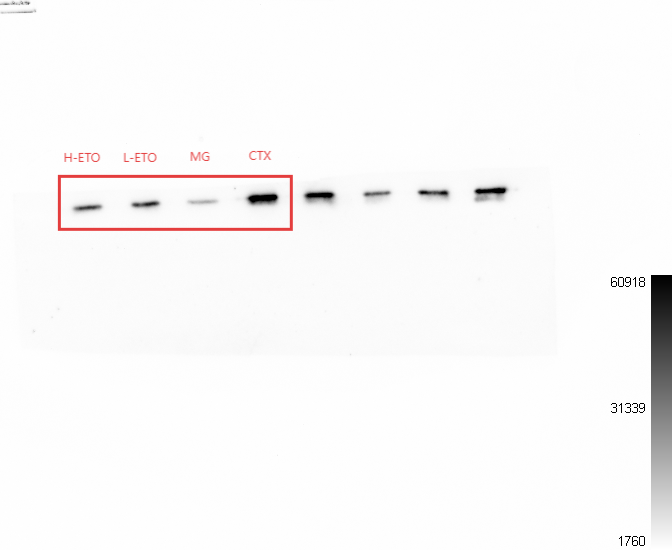


KF-kB

P-IKB

Caspase-3

IKB

BAX

Bcl-2

Supplement: Supplementary file 16 [file Supplementaryfile4.docx]

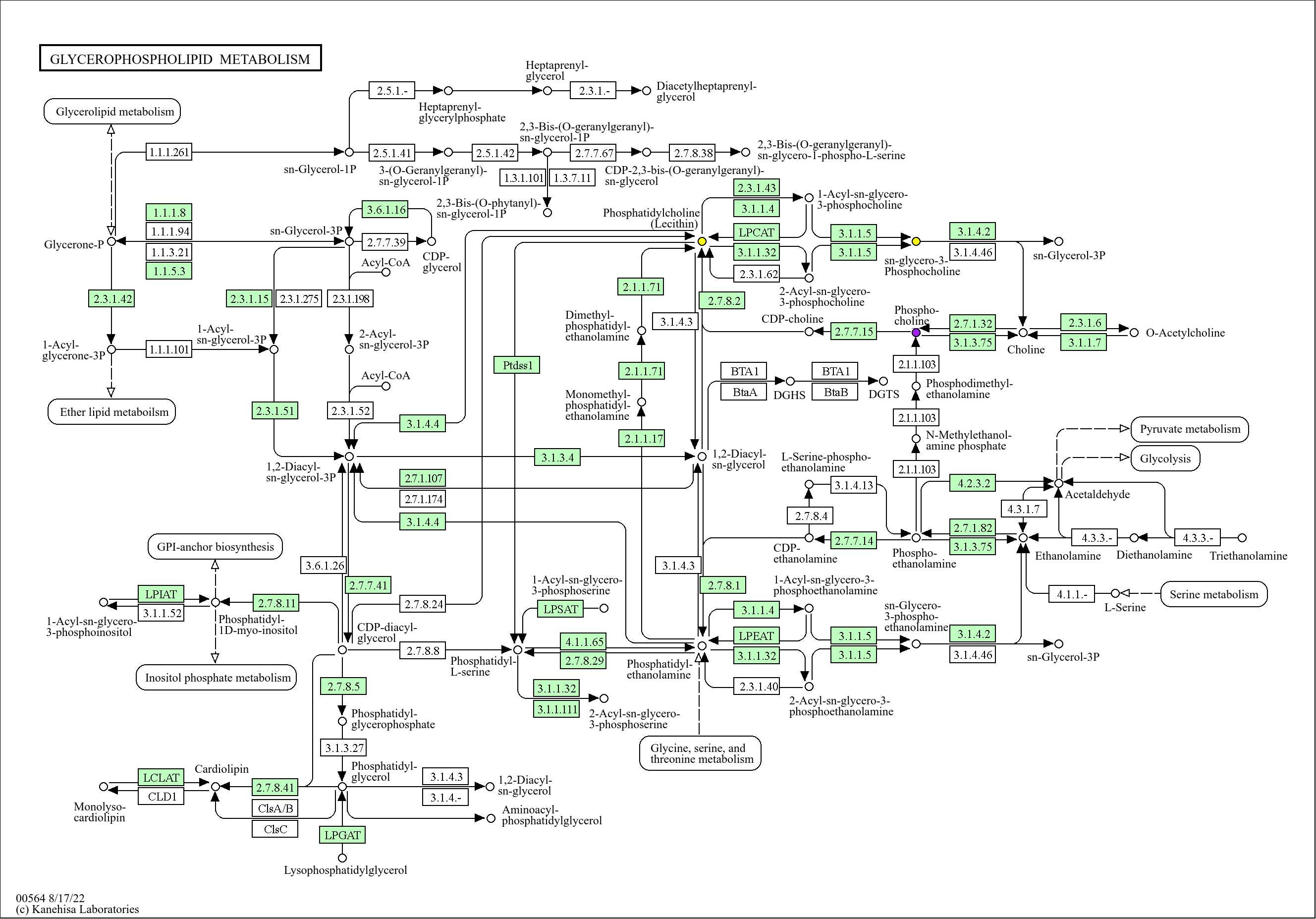

Supplement: Supplementary file 17 [file Image15.png]

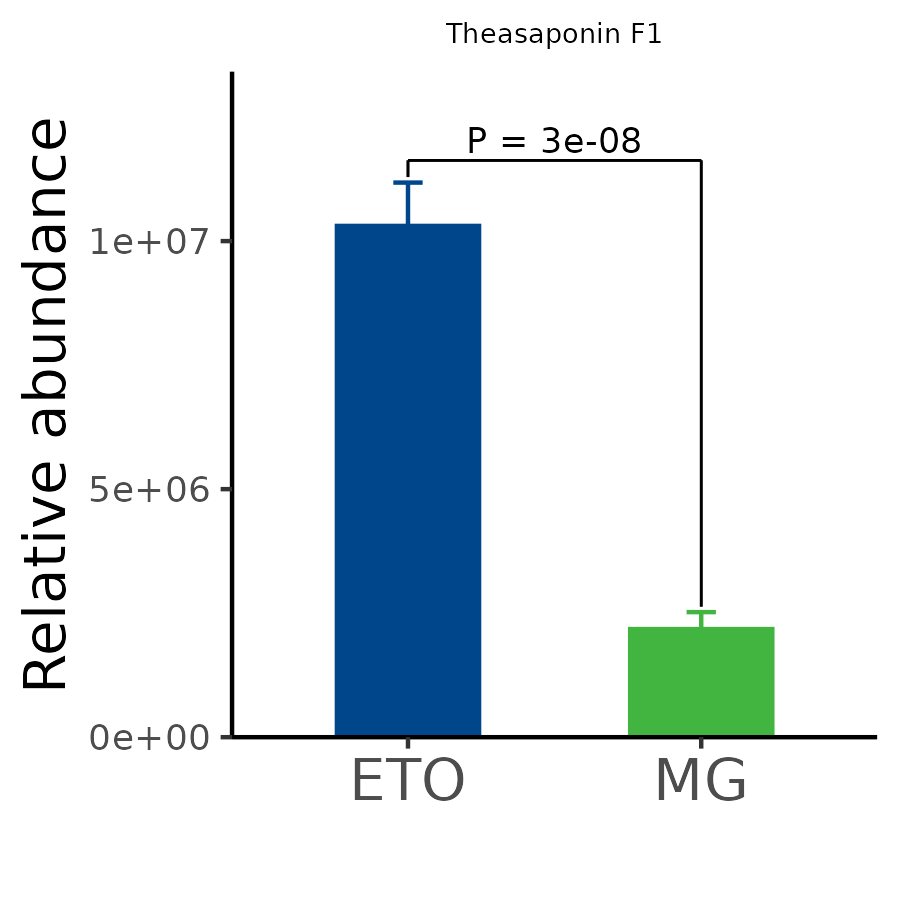

Supplement: Supplementary file 18 [file Image3.png]

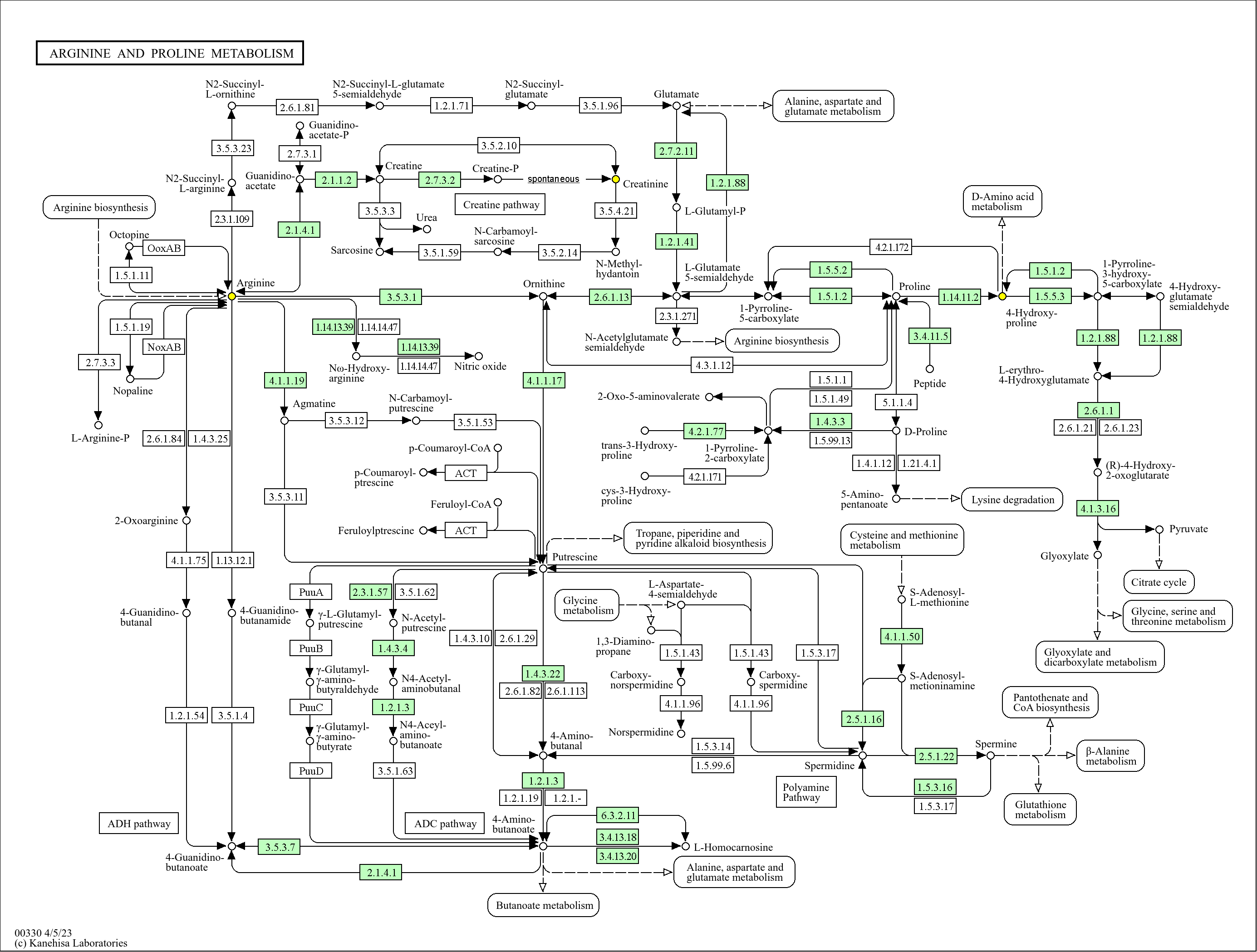

Supplement: Supplementary file 19 [file Image10.png]
