# Supplementary material for: Integrated gut microbiota and metabolomics analysis reveals the antitumor effects of ergosta-4,6,8(14),22-tetraen-3-one purified from the medicinal fungus pholiota adiposa in tumor-bearing mice
Source: Front Pharmacol. 2025 Dec 16;16:1653035. doi: 10.3389/fphar.2025.1653035 (PMC12748264; doi:10.3389/fphar.2025.1653035)

Ergosta-4, 6, 8 (14), 22-tetraen-3-one was characterized by ^1^H NMR (600MHz) and ^13^C NMR (125MHz) analyses recorded on the Varian Mercury NMR spectrometer equipped with superconducting magnets (Institute of applied chemistry, Changchun, China).

Pale yellow needle crystal (petroleum ether), mp 263-278℃, molecular formula C_28_H_40_O, ei-ms m/z:392[m]+. The chromatographic plate was detected by uv lamp and showed yellowish-green color at 365 nm. The color of iodine vapor (733mg). ^1^H-NMR (CDCl3, 600 MHz) δ: 5. 22 (1H, dd, J = 8. 0, 15.2 Hz, H-23), 6.05 (1H, d, d, J = 9.4 Hz, H-6), 5.64 (1H, s, H-4), 5.20 (1H, dd, J =8. 0, 15. 2 Hz, H-22 ), 1.05 (3H, d, J = 6. 6 Hz, H-21) , 0. 99 (3H, s, H-19) , 0.95 (3H, s, H-18), 0.93 (3H, d, J = 6.8 Hz, H-28) , 0. 84 (3H, d, J = 7.1 Hz, H-27) , 0.87 (3H, d, J = 8.1Hz, H-26); ^13^C-NMR (CDCl3, 125 MHz) δ: 200.1(s, C-3 ) , 122.4 (d, C-4) , 164.6(s, C-5) , 124.8( d, C-6) , 134.4(d, C-7) , 124.8(s, C-8) , 44.4(d, C-9) , 36.9(s, C-10) , 34.3 (t, C-1) , 34. 4 (t, C-2) , 19.7(t, C-11) , 35.7(t, C-12) , 44.1(s, C-13) , 156.3(s, C-14) , 25.5(t, C-15) , 27.9( t, C-16) , 55.8(d, C-17) , 19.7(q, C-18) , 16.8(q, C-19) , 39.4(d, C-20) , 21.4(q, C-21) , 135.6(d, C-22) , 132.9(d, C-23) , 43.0(d, C-24) , 33.2(d, C-25) , 20.2( q, C-26) , 20.1(q, C-27) , 18.1(q, C-28).


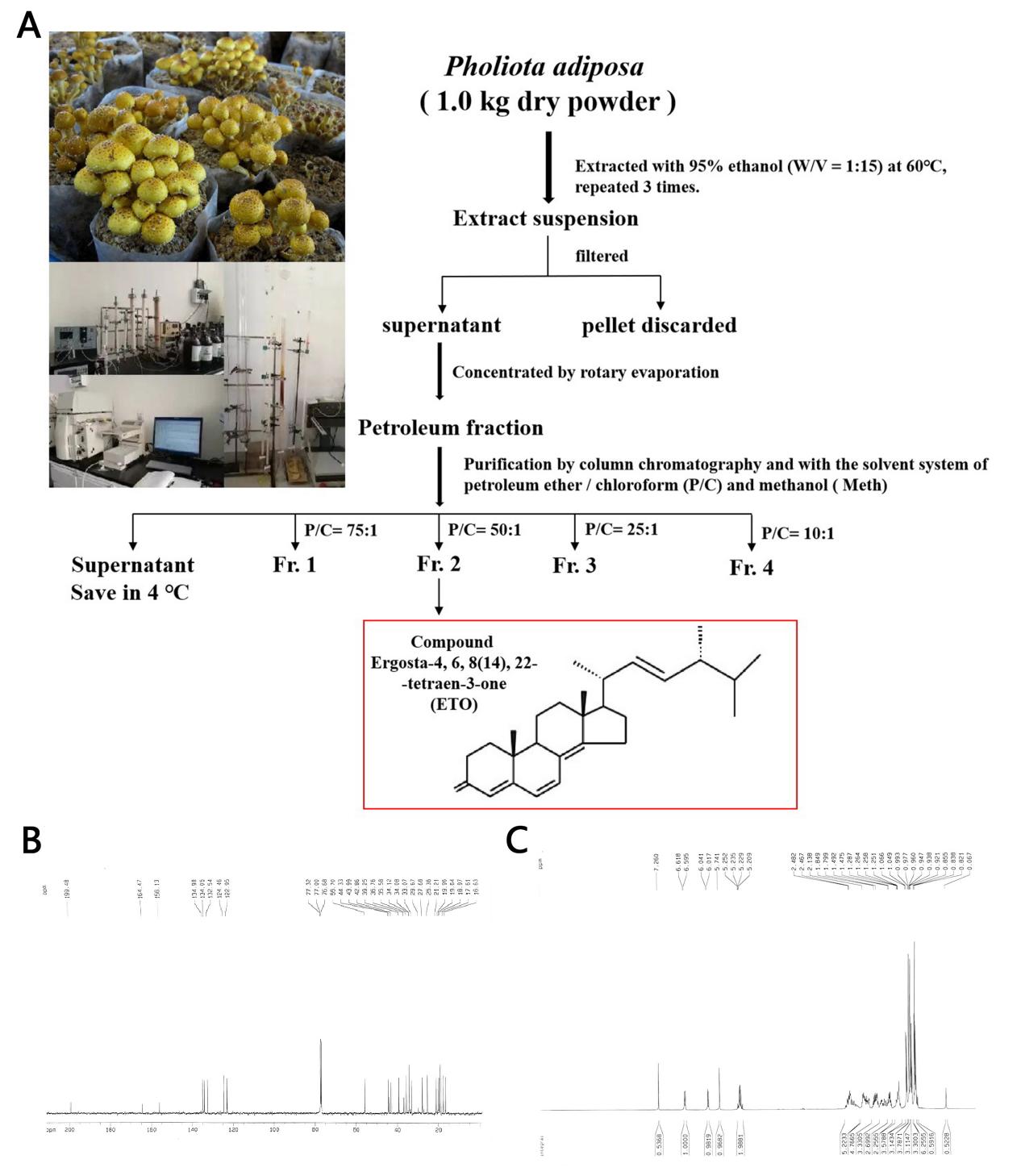

Supplement: Supplementary file 3 [file Supplementaryfile1.docx]
